# Allosteric interactions between RyR channels justify intracellular Ca^2+^ release of skeletal muscle in quantitative detail

**DOI:** 10.1085/jgp.202613968

**Published:** 2026-07-21

**Authors:** Eduardo Rios, Gonzalo Pizarro

**Affiliations:** 1 Department of Physiology and Biophysics, Section of Cellular Signaling, Rush University, Chicago, IL, USA; 2 Departamento de Biofísica, Facultad de Medicina, Universidad de la República, Montevideo, Uruguay

## Abstract

This modeling study explains the intracellular Ca^2+^ release that controls contraction of skeletal muscles of mammals as emerging from a statistical ensemble of couplons. Couplons are constituted by identical sarcoplasmic reticulum RyR1 channels, arranged in native “checkerboard” double-row pattern, with alternate channels (the V class) in physical contact with Ca_V_1.1 tetrads resident in the electrically excitable T-tubular membrane. Activation starts from these and propagates allosterically to the RyRs devoid of Ca_V_ contacts (the C class). Both activations are imposed by changes in free energy of channel states, derived from electrical work on the Ca_V_ mobile charges. The allosteric connections, resulting in reciprocal energy changes, are interlaced, so that the couplon becomes a highly reactive continuum where allosteric effects may propagate from end to end. A robust inactivation of C channels maintains this highly excitable device under graded voltage control. The model reproduces within the variance of experimental observations the cell level records of Ca^2+^ flux, including voltage dependence and kinetics, under multiple combinations of voltage clamp pulses, and accounts for the conditioning effects known as “quantal release” and “deterministic inactivation.” The simulations, which describe individual channel evolutions as stochastic Markov chains, are also consistent with the Ca^2+^ events recorded at the subcellular microdomain level as well as observations of coupled gating in bilayers. No explicit Ca^2+^ roles on activation or inactivation are required. The good model-vs.-experiment match enables inferences on mechanisms, their specializations in muscle tissues, and their variations in different taxa. It also encourages modular incorporation into more comprehensive models of cellular function.

## Teaser

The contraction of skeletal muscles is turned on by Ca^2+^ released from the cellular store by RyR channels in a quasi-crystalline array, half of which only have contacts with neighbor RyRs. Rios and Pizarro’s model assumes these channels to be controlled allosterically by their neighbors and matches quantitatively observations of Ca^2+^ release gathered over decades.

## Introduction

**Figure 1.**
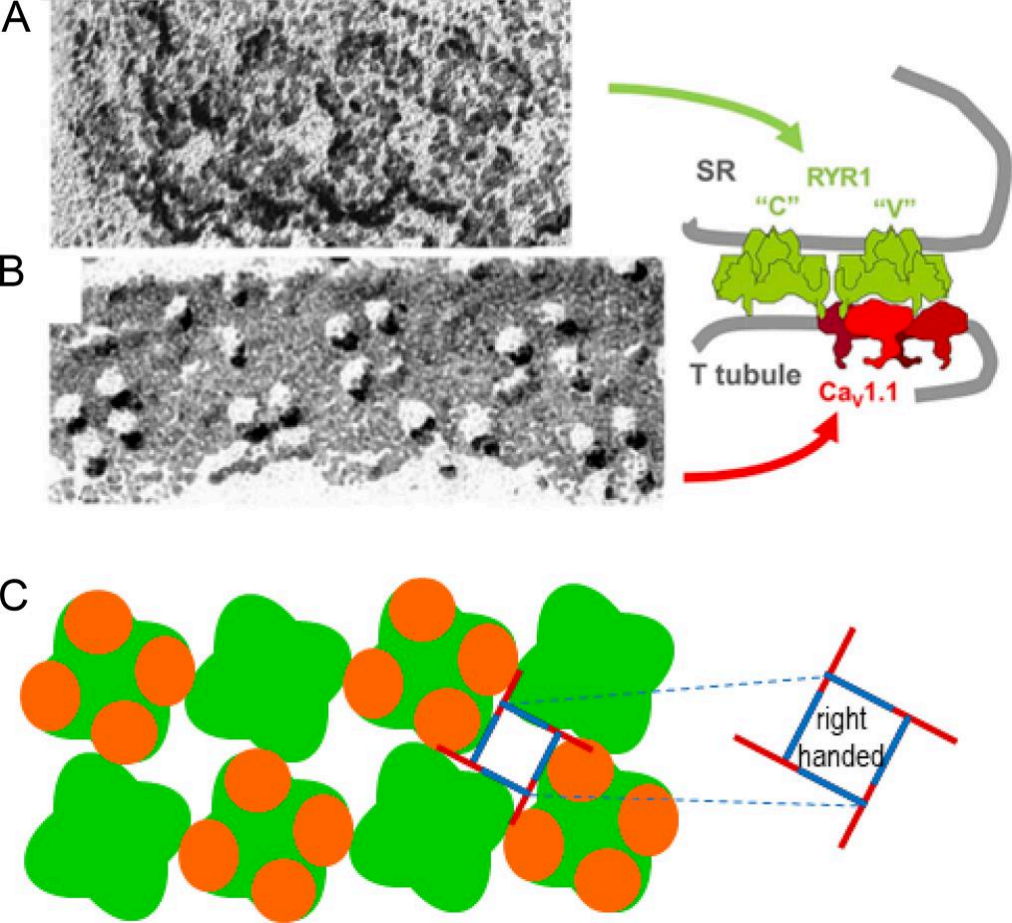
**Components of a junction between a transverse (T) tubule and an SR terminal cisterna in skeletal muscle. (A)** Freeze-dried junctional SR membrane from guinea pig showing RyR1 tetramers in checkerboard formation. **(B)** Tetrads of Ca_V_s in a freeze-fractured T tubule membrane from toadfish muscle, presented with the same orientation and magnification. **(C)** Canonical couplon, in which every other RyR is in contact with Ca_V_s (orange elements), thus constituting the “skipping pattern.” The diagram illustrates chirality or handedness, as viewed from outside the cell, in the way RyR tetramers make mutual contact. This orientation, called right-handed, has the inter-tetramer approaches occurring at the tetramers’ edge, to the right-hand side of every corner. Relabeled Fig. 1 of Ríos et al. (2019), which includes images from Block et al. (1988).

The contraction of striated muscles, skeletal and cardiac, is due to a mechanochemical reaction enabled by a fast increase in the concentration of free Ca^2+^ in the cytosol. Ca^2+^ is released from the sarcoplasmic reticulum (SR) via Ca^2+^ release channels (RyRs, which in mammalian skeletal muscle are of isoform 1) clustered in specialized junctional sub-compartments of the SR, the terminal cisternae. In “T-SR” or triadic junctions, the SR membrane is closely apposed to the membrane of transverse (T) tubules, which carries the action potential depolarization that signals the RyR1 channels to open. This command is transduced from the T membrane to the RyRs by voltage (V)-sensitive Ca^2+^ channels (Ca_V_1.1, also known as DHPRs). Structural aspects of these interactions are illustrated in Fig. 1. The set of contiguous RyRs in an SR junctional cisterna, together with the abutting Ca_V_s and associated proteins constitute a functional unit, the couplon (Stern et al., 1997).

The communication between DHPRs and RyRs in skeletal muscle is mechanical, a process termed depolarization-induced Ca^2+^ release, whereby conformational changes associated to the outward movement of electrically charged transmembrane helices (S4) of the voltage-sensing domain (VSD) of Ca_V_s cause conformational changes in the connected RyRs that open their pore.

This conformational interaction between voltage sensors and RyRs explains the gating of the Ca_V_-contacting channels (named “V” [Rios and Pizarro, 1988]), but not that of the RyR1 channels devoid of contact with Ca_V_s (named “C”), which constitute 50% of the total. The present study is an attempt to establish the mechanism of activation of this half of the RyR endowment. This is done via a quantitative model defined by the assumption that C channels are controlled allosterically (a term defined later) by V channels (the roman V designates channels, while the italic V represents transmembrane voltage). The model reproduces in quantitative detail multiple features of skeletal muscle Ca^2+^ release that have been revealed over many decades of experimentation and never fully explained.

The origin of the question addressed by this study can be defined with precision. In 1988, shortly after the nature of the voltage sensors for Ca^2+^ release had been proposed (Rios and Brum, 1987), Franzini-Armstrong and colleagues (Block et al., 1988) provided electron microscopic images of frozen-fractured T-SR junctions (Fig. 1) with momentous implications. They showed on the SR side of the fracture, RyRs forming a cluster with the previously established “checkerboard” pattern (Franzini-Armstrong and Nunzi, 1983), a quasicrystalline double row, and on the T membrane side of the fractured junction, tetrads of globular units also in a regular pattern. As Block and associates observed, they were of a size consistent with a Ca_V_1.1 (which at the time firmed their chemical identity); they also noted that the tilt of the tetrad with respect to the longitudinal axis of the couplon allowed a detailed one-on-one superposition on the individual protomers of the RyR1 homotetramers, while skipping every other RyR in a zig-zag or “skipping” pattern. As the authors argued, the detailed stoichiometric correspondence was indicative of a mechanical connection, as first proposed by Schneider and Chandler (1973), rather than a diffusible messenger-mediated communication.

As recently reviewed (Rios, 2026), all these suggestions have since been confirmed. Specifically, Cabra et al. (2016) for cardiac muscle and Chen and Kudryashev (2020) and Xu et al. (2024) for skeletal muscle confirmed that the mechanical contact is intricate, that it involves well-defined domains and that one tetrad of Ca_V_s only interacts with one RyR1 tetramer, the one it directly overlaps (This was not clear in the earlier structural studies, as the silhouette or contour of the tetrad exceeded that of an RyR tetramer, which left open the possibility of one Ca_V_ interacting with multiple RyRs). Thus, the question of activation of the C channels is now sharply defined: they cannot be directly controlled by the voltage-sensing Ca_V_s.

The question was addressed in the same year of 1988 by Rios and Pizarro, who proposed that the channels in contact with V-sensitive Ca_V_s were directly activated by them, while the intercalated RyR channels were opened secondarily in response to the Ca^2+^ emanating from the open channels (thus justifying the respective V and C labels). The proposal of activation by Ca^2+^ was followed by quantitative formulations that modeled or disputed it, combined with evolving evidence of a role of Ca^2+^ in the termination of Ca^2+^ release (Shirokova et al., 1996; Schneider and Simon, 1988; Simon et al., 1991; Jong et al., 1993; Jong et al., 1995a; Jong et al., 1995b; Jong et al., 1996; Pape et al., 1993; Pape et al., 1995; Pape et al., 1998, Stern et al., 1997, rev. by Ríos, 2018).

**Figure 2.**
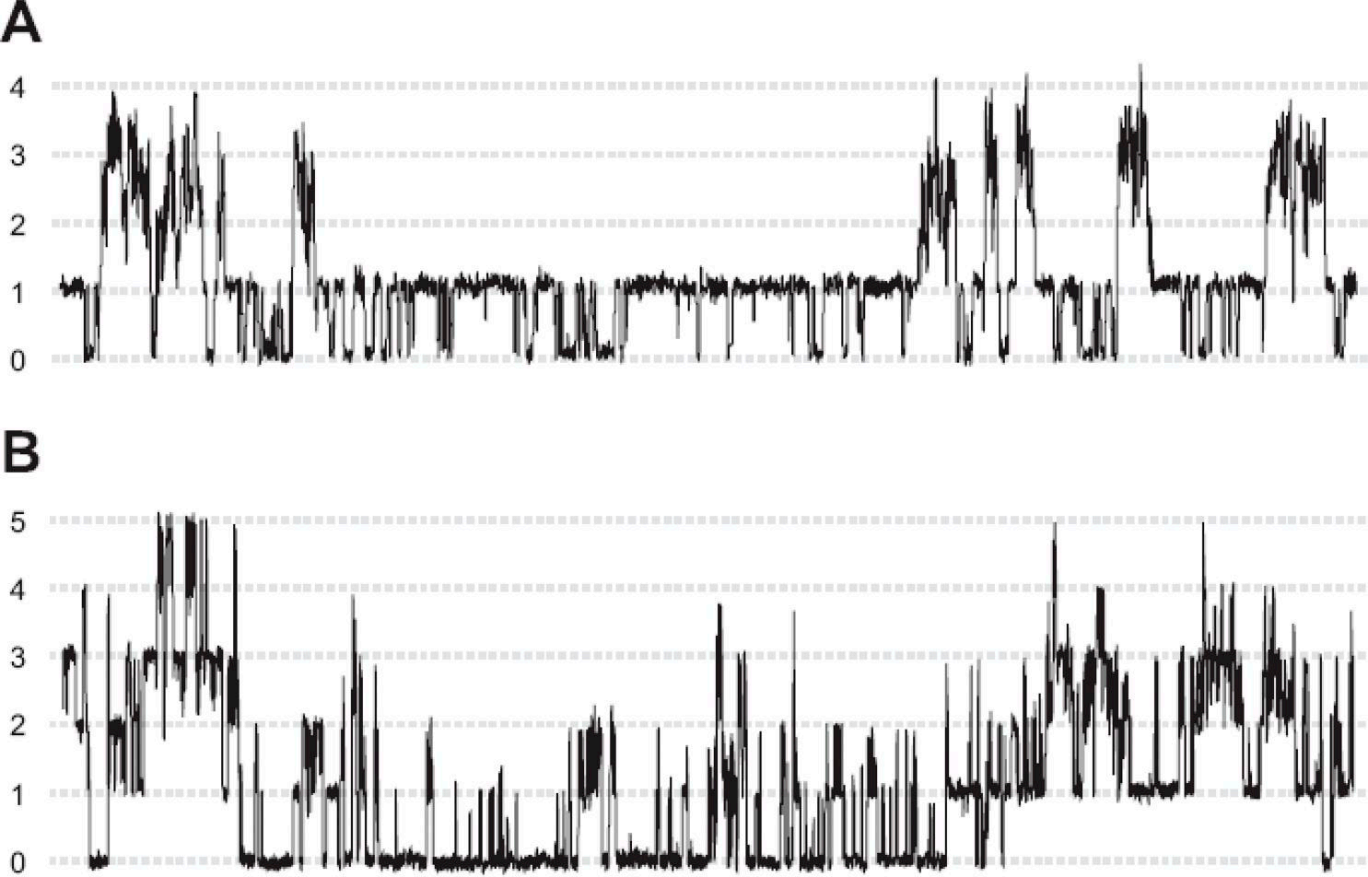
**Ca** ^**2+**^ **currents through RyR channels of rabbit skeletal muscle reconstituted in bilayers, exiting a *trans* compartment with ∼50 mM free [Ca** ^**2+**^ **].** Republished from Porta et al. (2012). **(A and B)** The authors interpret that the record in A arises from a set of four channels and those in B from one of five or six channels. Channels can be seen to open or close either individually or together in pairs, in what the authors call “partial coupling.” Records are compared with simulations in Figs. 19 and 20. Republication authorized by Prof. Julio Copello. Fig. 2 is reprinted with permission from *American Journal of Physiology*.

This pursuit of mechanism at the cellular level developed in parallel with the description of currents of RyRs reconstituted in lipid bilayers, which identified their susceptibility to activation by Ca^2+^ acting on the cytosolic side and, at higher Ca^2+^ concentrations, to inhibition and closing (Fill and Copello, 2002; Laver, 2018). At about the time that the roles of V and C channels were first discussed, Andrew Marx’s laboratory showed evidence of their allosteric interactions in bilayers, demonstrating “coupled gating,” first of RyR1s (Marx et al., 1998) and later of RyR2s (Marx et al., 2001). Albeit difficult to observe, the phenomenon was confirmed by other groups (rev. Rios, 2026), including a study of Porta et al. (2012), whose records will be taken as reference for the present simulations (Fig. 2). That allosteric coupling favors specifically channel opening is also supported by MD simulations showing that self-assembly of RyRs in checkerboard arrays occurs preferentially with channels in an open configuration (Xu et al., 2024).

The observation of coupled gating motivated a quantitative model of allosteric channel control for cardiac channels (Stern et al., 1999). Surprisingly, while other proposals for inter-RyR2 interactions have followed, e.g., Sobie et al. (2002) and Groff and Smith (2008), no formal model that includes inter-RyR1 allosteric coupling was proposed until a recent, pioneering attempt by Stephenson (2024).

The present modeling applies the physics introduced by Stern et al. (1999) (i.e., simulating the cell level Ca^2+^ flux as produced by a statistical ensemble of identical couplons at constant temperature), defines the couplons’ properties guided by their known structure (Fig. 1), and uses the streamlined procedure developed by Groff and Smith (2008) to simulate the many observations summarized below.

### The observations

The experimental findings that will be targets for simulation constitute a vast database, which spans the range from currents of individual channel in lipid bilayers to the macroscopic flux in whole-cell preparations. Crucial representative aspects of RyR1 activation are recalled here, reprinting images from published work by us and other laboratories.

Fig. 2, reprinted from Porta et al. (2012), illustrates allosteric coupling at the single channel level (channels from rabbit muscle). It shows that small groups of bilayer-reconstituted channels may couple their gating but do so variably and intermittently.

**Figure 3.**
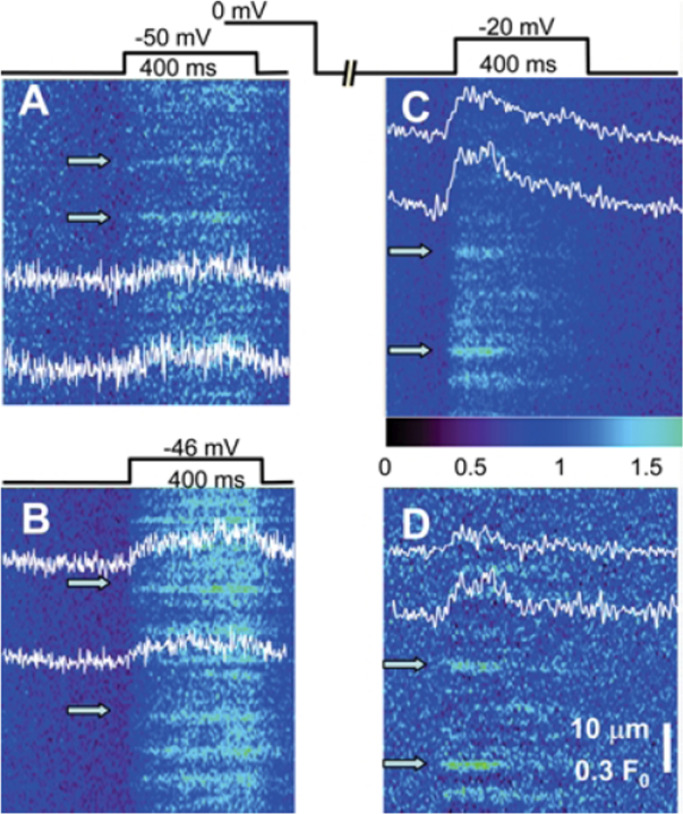
**Line scan images of Ca** ^**2+**^ **-monitoring fluorescence of fluo-3, elicited by depolarization in patterns shown at top in a myofiber from rat EDL—extensor digitorum longus—muscle (republished from Csernoch et al., 2004). (A and B)** In A and B, the events of Ca^2+^ release were elicited by voltage clamp pulses of very low voltage. **(C and D)** In C and D, the release system was partially inactivated by a previous depolarization, causing events elicited at higher voltages to be fewer and separate. The traces plot the fluorescence intensity at the sites marked by arrows. The increase over baseline (F_0_) was intermittent during the pulses and much lower than in spontaneous spark-like events seen in the side pools of the Vaseline-clamp device, where the fiber membrane is destroyed with detergent. Events in side pools are shown in Fig. 1 of the same article. Fig. 3 is reprinted with permission from *Journal of Physiology*.

An illustration of events at the single couplon level is in Fig. 3, reprinted from Csernoch et al. (2004). The observations on rat muscle inform a basic tenet of the present model: that individual skeletal muscle couplons response to depolarization is graded with voltage (by contrast with cardiac couplons, which tend to activate in all-or-none fashion, producing Ca^2+^ sparks).

**Figure 4.**
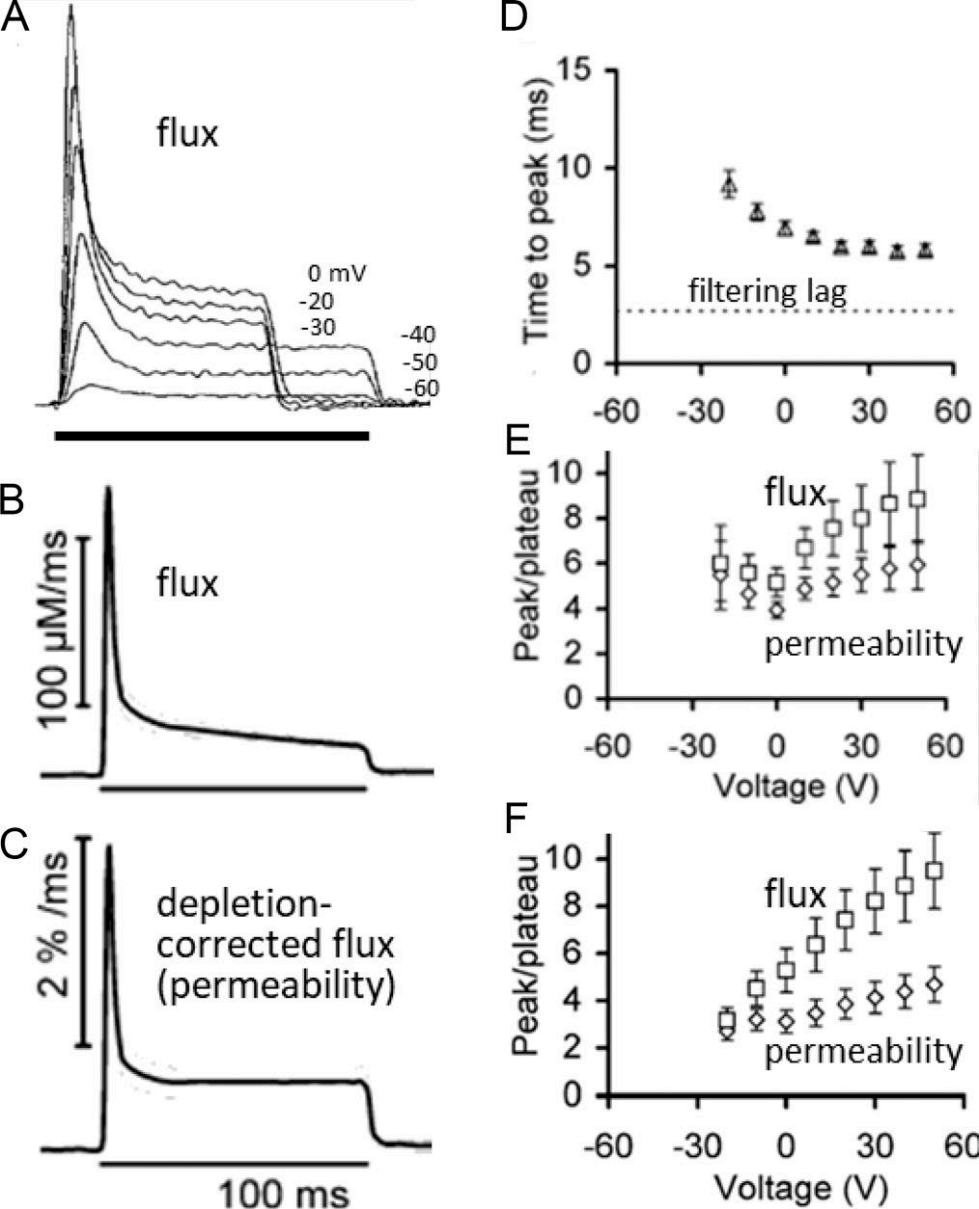
**Ca** ^**2+**^ **release flux and its correction. (A)** Ca^2+^ release flux derived from Ca^2+^ signals of ApIII in a rat EDL myofiber under voltage clamp; flux rises to a peak and decays to a stage that at low voltages is steady and at higher voltages decays slowly. **(B)** Flux in a speed-optimized clamp setup. **(B–F)** Mouse interosseus fibers perfused with Fura-2 (B, C, and F) or Fura-FF (D and E). **(C)** Correction for depletion yields a signal proportional to release membrane permeability. **(D)** Times to peak permeability from Fura-FF record. **(E and F)** Peaks of flux and corrected flux or permeability vs. voltage. Partial reproductions of Fig. 3 in Shirokova et al. (1996) (A) and of Fig. 9 in Ursu et al. (2005) (B–F), with labels added. Fig. 4 reprinted with permission from *Journal of Physiology*.

**Figure 5.**
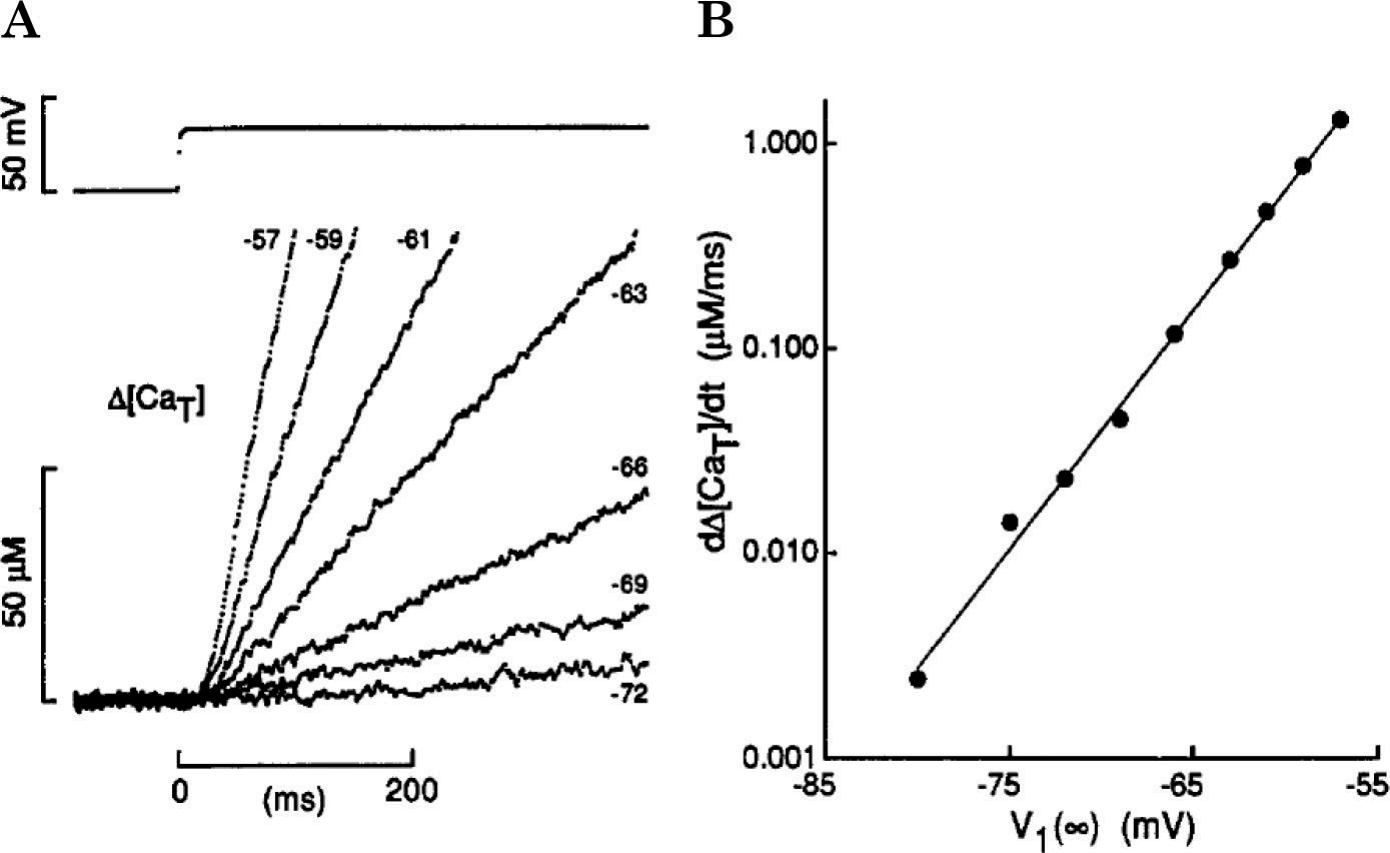
**Evolution of total released Ca** ^**2+**^ **in a voltage-clamped frog myofiber. (A)** Total released Ca^2+^ vs. time. Ca^2+^ concentration was measured with Fura-2 and total released was quantified as the increase in total cytoplasmic Ca^2+^ (Δ[Ca_T_]), mostly bound by EGTA in the experiment. Flux is the rate of change of the records in A (dΔ[Ca_T_]/dt). **(B)** Semilog plot of flux vs. applied voltage, with a slope corresponding to an *e*-fold increase every 3.73 mV. Reproduced Fig. 14 in Pape et al. (1995).

**Figure 6.**
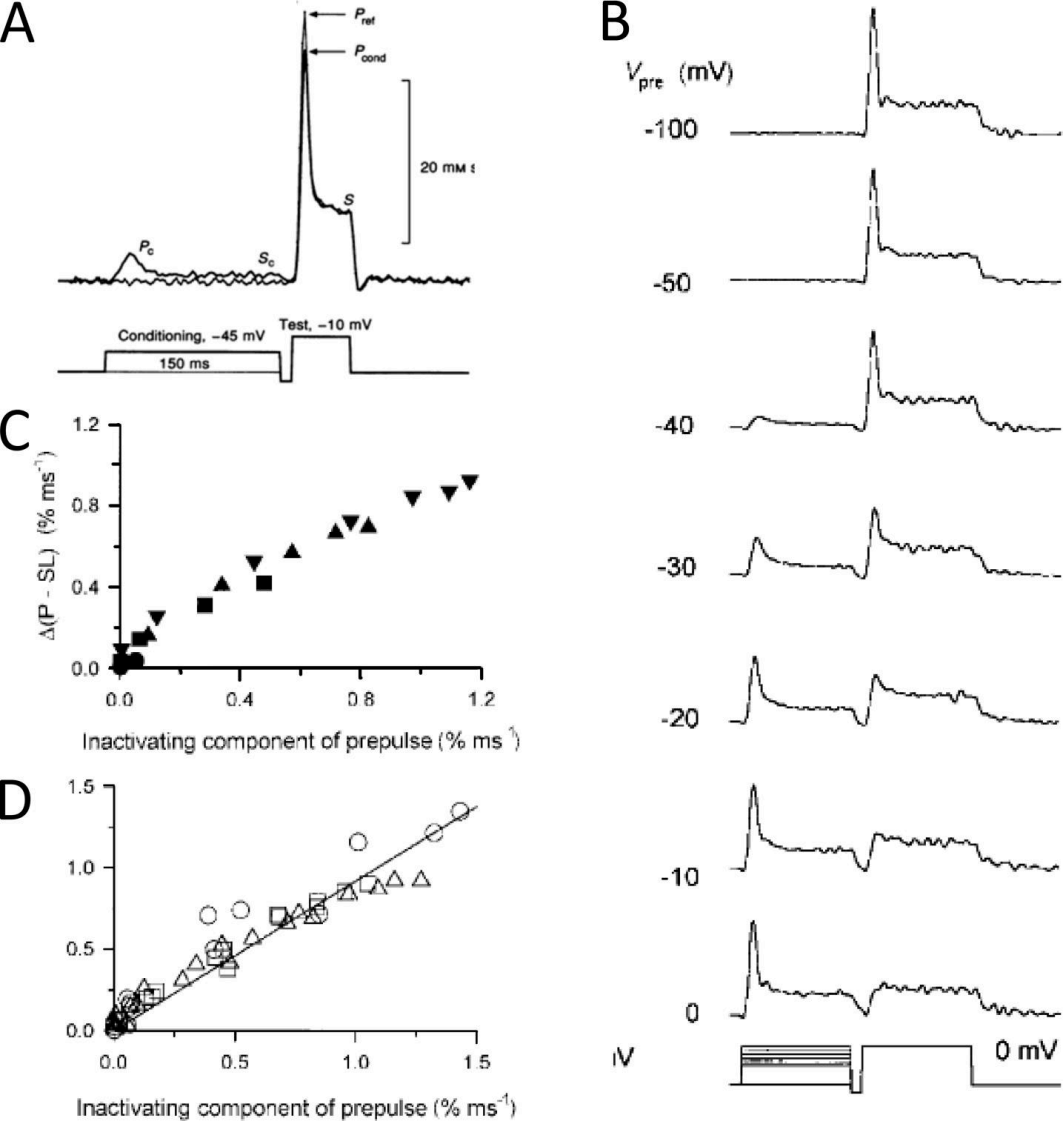
**Quantal Ca** ^**2+**^ **release and deterministic inactivation. (A)** Superimposed records of flux elicited by the two-pulse patterns shown in a voltage clamped frog myofiber. The presence of a conditioning pulse reduces the peak of the flux elicited by the large test pulse *P*_ref_ to a value *P*_cond_. The difference *P*_ref_-*P*_cond_, called “suppression,” is approximately equal to “decay” of flux (*P*_c_-*S*_c_) elicited by the conditioning pulse. Conditioning does not change the steady value S reached at the end of the test pulse. Reproduced from Pizarro et al. (1997). **(B)** A set of fluxes elicited by pairs of pulses in rat EDC muscle; a large test pulse follows a conditioning pulse of variable amplitude. **(C)** The values of decay in the flux elicited by the conditioning pulses of panel B, plotted vs. the suppression induced in the test flux. **(D)** Replications of the same plot in different individuals. From Szentesi et al. (2000), authorized by Péter Szentesi and László Csernoch. Fig. 6 reprinted with permission from *Journal of Physiology*.

**Figure 7.**
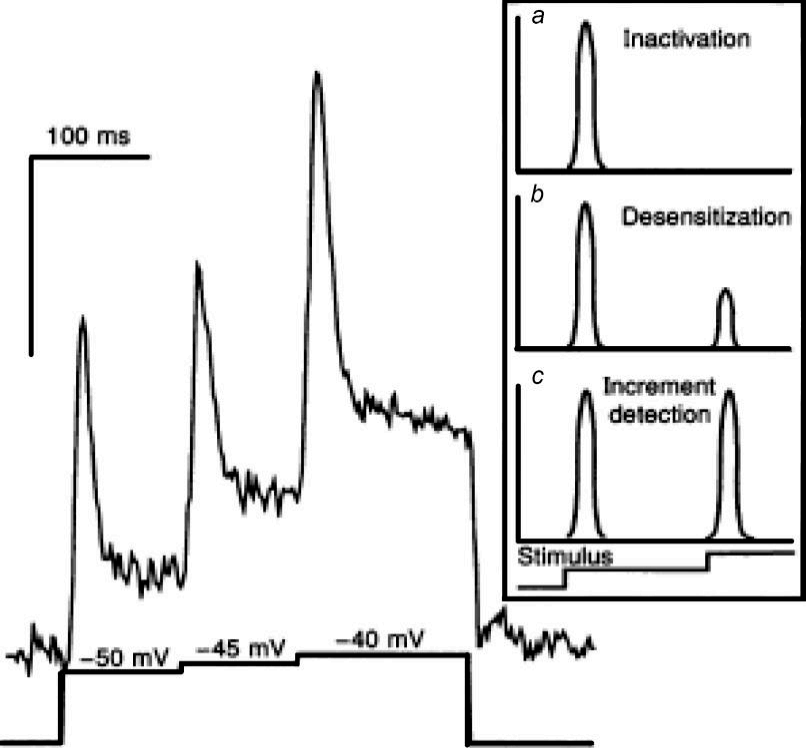
**Increment detection in *Rana*.** Ca^2+^ flux in response to the three-step increase in voltage shown. Inset, three patterns of transient response to two steps of stimulus intensity (from Meyer and Stryer, 1990): *a*, “classical” inactivation, if complete, results in no response to a second step; *b*, desensitization (also known as adaptation), resulting in a smaller response to the second step; *c*, the ideal increment detection, with responses proportional to stimulus increment. From Pizarro et al. (1997).

**Figure 8.**
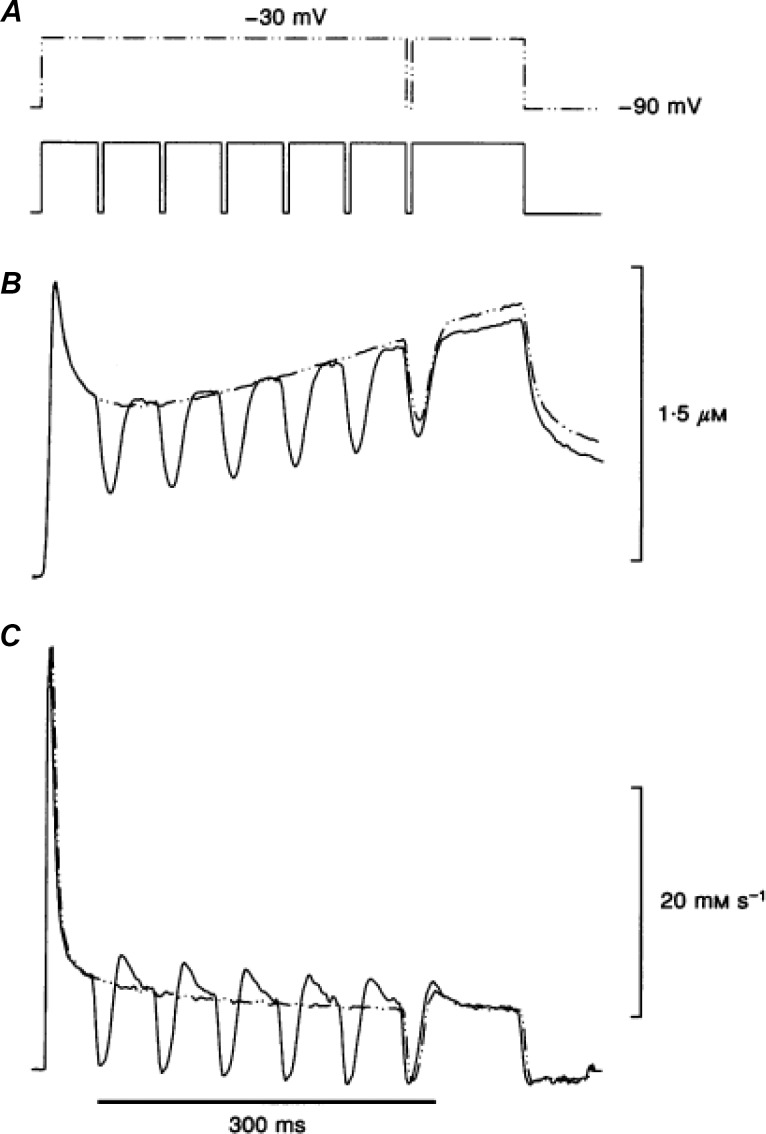
**Calcium transients and release flux activated by a train of pulses.** **(A–C)** Superimposed cytosolic [Ca^2+^] transients (B) and derived depletion-corrected Ca^2+^ release flux (C) elicited by the two pulse patterns in A. The nearly complete (91%) suppression of release in the pulses that follow the first one is not altered in spite of the large variation in cytosolic [Ca^2+^]. Reproduced from Pizarro et al. (1997). Fig. 8 reprinted with permission from *Journal of Physiology*.

The properties of the Ca^2+^ flux of fast skeletal muscle myofibers, recorded under voltage clamp, are illustrated with Figs. 4, 5, 6, 7, and 8. Fig. 4 A shows Ca^2+^ flux in a fast twitch muscle fiber from the rat, with a characteristic early rise to a peak, followed by a fast decay to a more slowly decaying phase. Ca^2+^ flux is a function of the gradient of (Ca^2+^) across the SR membrane and the collective permeability of the set of channels where it flows. Soon after the initial development of flux measurement (Baylor et al., 1983; Melzer et al., 1984), a method was devised to remove the variations in driving force from the measured flux; the actual output being a “depletion corrected flux” proportional to permeability (Schneider et al., 1987; rev. Hernández-Ochoa and Schneider, 2012). It is illustrated here with records Fig. 4, B and C, obtained with a technique optimized for clamp speed in Werner Melzer’s lab (Ursu et al., 2005). Throughout this article, the output of the model, referred to as flux (F), will be simulations of the depletion-corrected version.

Whereas the bulk of the early experimentation was done on members of the frog genus *Rana*, the work with mammalian muscles (largely mouse and rat) only started with a study by Garcia and Schneider (1993). Unlike mammals, whose couplons only have RyR1, muscles of nonmammalian taxa, including *Rana*, have RyRs of isoforms 1 and 3 (see Perni et al., 2015). Their Ca^2+^ release includes Ca^2+^ sparks, a paradigm of Ca^2+^-induced Ca^2+^ release (CICR), which requires RyR3 (reviewed by Ríos [2018]). The present study models specifically Ca^2+^ release in mammals (which as shown in Fig. 3 does not include sparks under physiological conditions). It accordingly assumes a couplon constituted by a homogeneous set of RyR1 channels; therefore, its output is compared with flux recorded in muscle of rats or mice. Some phenomena, however, including flux at exceedingly low voltages and aspects of release inactivation and recovery, have been studied only in *Rana*. Those observations will be used as reference where the work with mammals is lacking.

#### Observations of kinetics and voltage dependence

##### Kinetics

The (depletion-corrected) flux elicited by a voltage clamp pulse features an early peak followed by a stage steady for the duration of the pulse. The typical waveform in rodents fast-twitch myofibers is illustrated in Fig. 4. We will take the set of fluxes derived by Ursu et al. (2005) in mouse muscle as target for quantitative simulation of the “fast” aspects of the phenomenon (the magnitude of the transient stage and the time to peak) because of the quality of the voltage clamp and care for reproducibility in this study.

The records reveal spread in the measurements, likely due to both individual variation and technical error (compare for instance in Fig. 4 the permeability ratios “Peak/plateau,” which differ by factors of two with the two dyes used). Our modeling dealt with this issue by aiming for quantitative properties in the mid-range of the variation.

Other kinetic features include the characteristic times of decay after the peak. Because these “slower” features are less sensitive to clamp speed, their measures were taken from both the records of Ursu et al. (2005), and the detailed studies of László Csernoch’s group on rat muscle (Szentesi et al., 2000; Szentesi et al., 2004), with an example in Fig. 6.

##### Voltage dependence

The dependence of Ca^2+^ flux on membrane voltage (V) is graded and sigmoidal, with both peak ($F_{P}$) and steady ($F_{S}$) levels increasing from fully closed to saturation in a range of ∼100 mV. Gradation with voltage prevails at the whole-cell as well as the single couplon level (as shown by Shirokova et al. [1998]; Csernoch et al. [2004], cf. Fig.3).

The parameters of the dependence of $F_{P}$ on V taken as reference are those determined in mouse by Ferreira Gregorio et al. (2017). In their study, peak flux increased sigmoidally with voltage, from a holding potential of −90 mV to saturation at about +10 mV. The dependence (on average over multiple replications in different murine strains) was fit with a “Boltzmann” function

$$F_{P}=F_{\max}{\left(1+e^{-\left(V-\bar{V}\right)/\kappa }\right)}^{-1}$$ (1)

with mid-voltage $\bar{V}$ = −39 mV and “slope factor” κ = 8.9 mV.

(While Shirokova et al. did not report a Boltzmann fit of their measurements in rat muscle [Fig. 4 A], the voltage at half-maximum of the peak flux was −36 mV [determined, by us, on their Fig. 5 A].)

A critical aspect of the V dependence of activation is revealed at very low voltages in frog muscle. Within the range of depolarizations between the holding potential, −90, and −60 mV, the flux lacks a peak, rising monotonically to a steady value. This was first demonstrated by Pape et al. (1995) for frog muscle, with records reproduced here as Fig. 5. Because the net flux elicited at these voltages is very low, the authors chose to show its integral over time, which is equivalent to the net increase in total cytosolic calcium concentration (Δ[Ca_T_]). The slope of these near rectilinear traces (i.e., the flux) is proportional to an exponential function of V—the limiting form of Eq. 1 for large negative V. As the authors pointed out, the fact that flux is independent of time and exponentially dependent on voltage reflects a simple activation process inconsistent with feedback via CICR or another cooperativity. Although a study with this level of precision has not been carried out in mammals, release flux waveforms elicited in mouse muscle at low voltages have a proportionally smaller peak or none at all (e.g., Fig. 6 B). The merit of any model must include matching these features in the limit of low voltages.

#### Conditioning: The “quantal” property of calcium release

When two depolarizing pulses are applied in close sequence, the flux elicited by the second pulse (“test”) is altered by reduction of the transient phase, partial, or total depending on the magnitude of the “conditioning” pulse, as shown with Figs. 6, 7, and 8. This conditioning, attributed to the same inactivation process that determines the fall of the flux from its initial peak, has two simple properties: it does not affect the steady phase of release at any conditioning voltage, and it recovers at the resting potential with an approximately exponential time course of time constant measured in mice by Sárközi et al. (2000) at 110 ms on average. This recovery occurs on an entirely different time scale than the voltage-dependent inactivation of Ca_V_1.1 (which takes seconds, e.g., Brum and Rios, 1987) or the time-dependent refilling of the SR after depletion (which takes tens of seconds, as shown, for example, by Schneider et al. [1987]; Fig. 4). Additionally, the inactivation phenomenon has multiple peculiar features, referred to collectively as “quantal” (Pizarro et al., 1997) or “deterministic” (Szentesi et al., 2000). The term “Quantal calcium release” was coined by Muallem et al. (1989) to name an observation in pancreatic acinar cells, extended later to other cells (Bootman, 1994): Ca^2+^ release induced by submaximal concentrations of inositol trisphosphate cannot completely deplete the internal calcium stores. The initial explanation for this surprising feature invoked different storage compartments with channels of different sensitivities to the agonist (Ferris et al., 1992). Right or wrong, this ‘heterogeneous model’ works as definition of quantal Ca^2+^ release; namely, release that depends on agonist concentration and time, as if resulting from the activation of channel subsets with different sensitivities to the agonist. Substituting voltage for the agonist, it applies to calcium release in skeletal muscle, as shown by Pizarro et al. (1997) for the frog and Szentesi et al. (2000) for the mouse. Both studies ruled out the possibility of a heterogeneous set of channels with different sensitivities and explained the observations assuming that only and all the channels that opened segued to inactivation. Pizarro et al. called this type of inactivation “fatal”; Szentesi et al. called it “deterministic”.

A defining quantal feature is illustrated in Fig. 6 A: the *decay* in the conditioning flux, difference between its peak (P_c_) and steady values (S_c_), is approximately equal to the *suppression* of the test response, the difference of peaks, P_ref_ − P_cond_, between an unconditioned reference and the conditioned one. This near equality is observed regardless of conditioning and test voltages (e.g., Fig. 6 B reproduced from Szentesi et al. [2000]). The typical relationship between decay in conditioning releases of variable voltage and the suppression induced in a large test is illustrated in Fig. 6 C. The slight divergence from equality, first in excess of equality, turning to a deficit at higher voltages is reproducible in experimental replications, e.g., Fig. 6 D.

Quantal release has other manifestations, useful physiologically (Pandol and Rutherford, 1992), and important here as additional features that any model should match. They have only been explored in frog muscle, but given the similarities already revealed by the study of Szentesi et al. (2000), they are likely present in mammalian muscle and will be targeted in the present simulation. One is “increment detection,” first described by Hajnóczky and Thomas (1994) and illustrated with Fig. 7. The inset, from Meyer and Stryer (1990), sketches responses to two successive incremental applications of an agonist: (1) illustrates full inactivation by the first application, (2) adaptation, and (3) increment detection. As the experimental trace from frog muscle by Pizarro et al. (1997) shows, voltage-induced Ca^2+^ release has the property of increment detection. It is expected of any system with quantal release and will be sought in the model output.

One more record from frog muscle is reprinted in Fig. 8: Ca^2+^ release flux in response to a train of large pulses separated by brief repolarizations. The traces show that the suppression in every pulse after the first one is similar and nearly equal to the decay in the first pulse (91% on average). The top records also show that this nearly complete suppression is achieved in spite of a large variation of the increase in the Ca^2+^ concentration at which it is experienced, from 0.4 µM in the first conditioned transient to ∼0.75 µM in the last one. This observation shows that, within limits, the global calcium concentration changes are irrelevant to the inactivation process. Additional evidence includes the requirement of hundreds of micromolar [Ca^2+^] to inactivate channels in bilayers (Ma et al., 1988; Tripathy and Meissner, 1996) and the lack of effects in frog muscle fibers of photorelease-induced increases in [Ca^2+^]_cytosol_ to micromolar levels (Hill and Simon, 1991).

### Theory

The present model simulates cell-level Ca^2+^ flux as emerging from an isothermal-isobaric statistical mechanical ensemble of couplons, for which Gibbs free energy is the appropriate work potential (strictly, an NPT ensemble; e.g., Pathria and Beale, 2011, chapter 7). It requires theories of allostery, activation by voltage and inactivation.

Two dualities constrain the model. The first one is structural. In the couplon of mammals there is only isoform 1 of RyR but two distinct classes, V and C, by virtue of their contact with Ca_V_1.1. On this basis, we assume that V channels are acted upon by voltage sensing Ca_V_s, while C channels are not. Because CICR does not operate physiologically in mammalian skeletal muscle (reviewed by Ríos [2018], with confirming evidence in Kobayashi et al. [2025]), the model assumes that C channels are controlled allosterically by the neighboring V channels.

The other duality is functional, the presence of two kinetic phases in the flux waveform: a “peaky” transient $F_{P}$ and a steady stage

$$F\left(V,t\right)=F_{P}+F_{S}$$ (2)

The model must reconcile the two dualities. To start, the flux F(V,t) will also be split according to their origin as

$$F\left(V,t\right)=F_{V}+F_{C}$$ (3)

In principle, two kinetic phases could be present in the flux through both V and C channels

$$F_{V}=F_{V,S}\left(V\right)+F_{V,P}\left(V,t\right)$$ (4)

$$F_{C}=F_{C,S}\left(V\right)+F_{C,P}\left(V,t\right)$$ (5)

where the subindexes identify origin (V or C) and kinetics (P or S) in the currents.

Because there is no information about the four Fs in Eqs. 4 and 5, we made the simplest assumptions possible (as in Rios and Pizarro [1988]), namely that only C channels inactivate, and can do so completely. The hypotheses eliminate $F_{V,P}$ and $F_{C,P}$. In consequence, Eq. 3 can be written more explicitly as

$$F\left(V,t\right)=F_{V}\left(V\right)+F_{C}\left(V,t\right)$$ (6)

Put simply, the steady component courses via V channels and the transient component via C channels. That channels V do not produce a transient ($F_{V,P}=0$) and channels C do not contribute to the steady phase ($F_{C,S}=0$) are crucial assumptions with ramifications for the physics of the model. (In Appendix C, we explore the alternatives and show their inability to reproduce the observations within the parameter space tested.)

A primary concern of the theory is with the scale of the transients. The maximum of peak flux, reached in depolarizations to positive transmembrane voltages, is ∼200 mM/s (e.g., Ursu et al., 2005). This measure is consistent with the known density of release channels, provided that most are activated. Thus, for the model to be consistent with this absolute scale of currents, most channels in the model will have to contribute to flux at high voltages. It will be shown that the present simulations satisfy this requisite.

As remarked before, and illustrated with Fig. 3, release flux is graded with voltage at the couplon level. This observation motivates a defining hypothesis of the present study: that the significant “macroscopic” features of Ca^2+^ release (i.e., those measured at whole-cell level) are all featured in the flux of individual couplons. This premise streamlines the procedure, as it removes the need to simulate sets of different couplons but is also severely constraining, as the array of functional features must be reproduced by a single model couplon, with its limited number of channels, which only affords a small number of parameters. To demonstrate that significant features of the model are not missed by focusing on one couplon size, Supplement 1 presents simulations with couplons of different numbers of channels.

In mice, large couplons, the ones expected to best represent the full repertoire of properties, have no more than 1 µm in length. Consequently, the model couplon here comprises 60 channels—a double row of 30 V and 30 C channels—which span a length of 0.8–9 μm.

The basic output of the model is the joint flux through 30 V and 30 C channels. As the absolute value of the flux is not a concern, flux is reported with no dimensions; that is, the flux through an open V channel is adopted as unit.

The total flux will consist of a non-inactivating $F_{V}$ component from V channels, with a maximum value of 30, plus an $F_{C}$ through 30 C channels, subject to inactivation, with a maximum value of 30 multiplied by the ratio of single channel fluxes $R_{C/V}$. Surprisingly and necessarily, $R_{C/V}$ must be >1; indeed, because the ratio Peak/Steady reaches values of four or greater at high V (e.g., Fig. 4 B), the numbers of C and V channels are exactly equal, and all or most activate at high V, the single channel flux ratio must be at least four. The optimal fit required a value of five, therefore

$$F\left(V,t\right)=n_{V}\left(V\right)+5n_{C}\left(V,t\right)$$ (7)

where $n_{V}$ and $n_{C}$ represent the numbers of open V and C channels. Therefore, the Fs in Eq. 6 are simple multiples of the numbers of open channels: $F_{v}=n_{V}$ and $F_{C}=5\;n_{C}$.

(Eq. 7 is a useful approximation. Strictly, because the transition rates that define the time course are finite, $n_{V}$ depends on time as well as voltage. But, in contrast with $n_{C}$, its changes are monotonic and fast, between levels defined by V only.)

#### Allostery

The allosteric interactions are treated pairwise. The approach starts from a state diagram of the channel, which in the absence of inactivation is just

$$C\overset{k^{+}}{\underset{k^{-}}{⇄}}O$$ (8)

**Figure 9.**
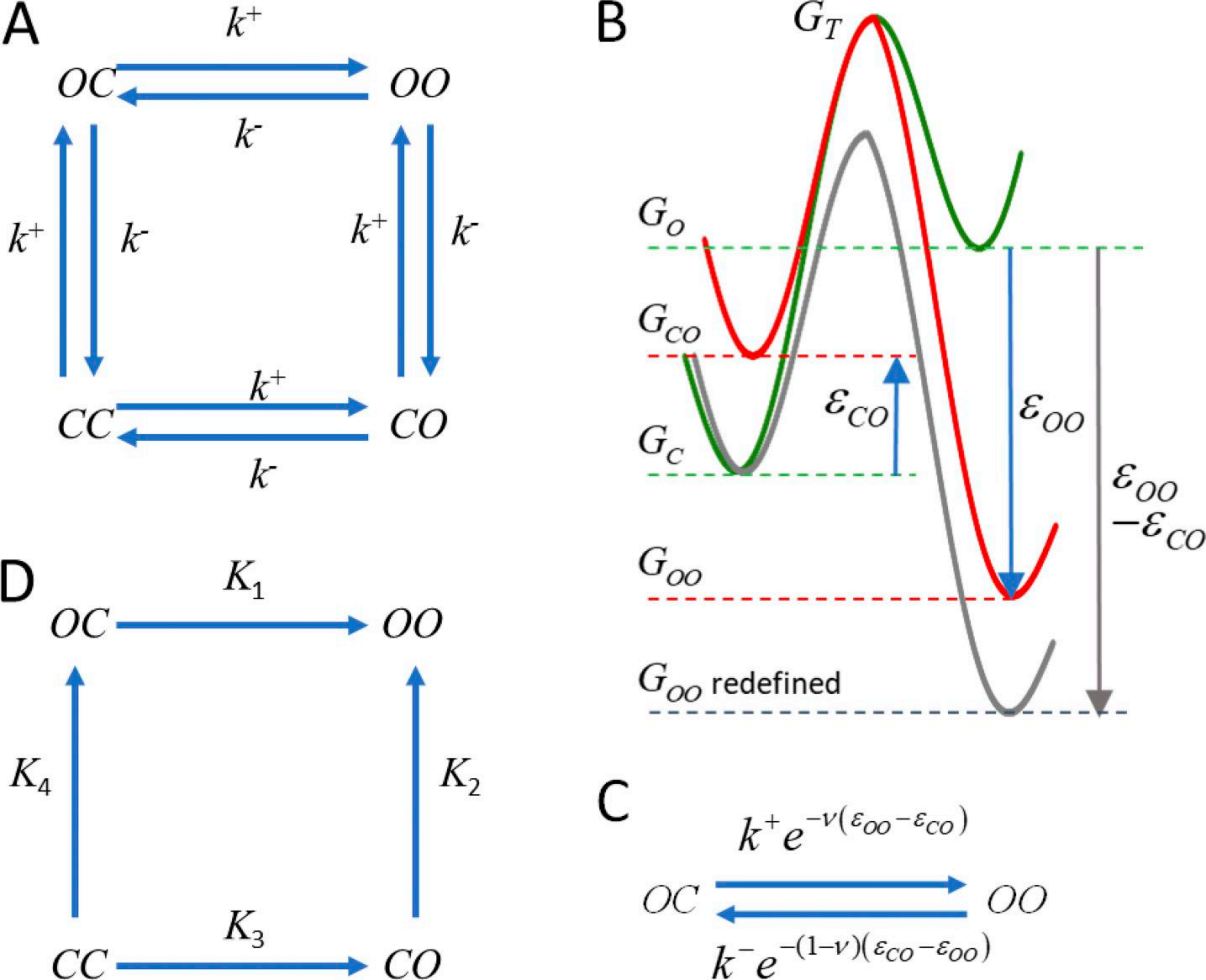
**Gating of independent and allosterically coupled identical channels. (A)** The transitions of two channels gating independently. **(B)** Assumed effects of coupling, in a transition rate theory formulation. The curve in green depicts Gibbs free energy along the transition path of a channel gating independently. In red is the curve modified by coupling to an open channel. In grey is a shifted version of the modified curve, corresponding to a shifted definition of energies, which simplifies the algebra and clarifies the allosteric effects without altering the physics (see note in text). **(C)** How the two “native” kinetic transition rates, $k^{-}$ and $k^{+}$, are modified by the interaction with an open channel. **(D)** Illustrates a requirement for microscopic reversibility, namely $K_{1}K_{4}=K_{2}K_{3}$, the implications of which are developed in Box 1.

The Gibbs free energies of a channel in the open or closed state are allosterically altered by neighbors pairwise, as illustrated with Fig. 9. Diagram A represents a pair of identical two-state channels gating independently. The transitions between the four possible states of the pair are governed by the two rate constants of the identical channels, the ratio k+k‐ of which is the equilibrium constant K of the gating reaction, proportional to an exponential of the free energy difference between the two states. Diagram B illustrates the energies in conventional Eyring rate theory format (Eyring, 1935; Truhlar et al., 1996). In green trace is the hypothetical free energy (G) along the transition coordinate between a channel C and O states; in this representation, $k^{+}$ and $k^{-}$ are, respectively, proportional to the exponentials of (minus) the barrier heights $G_{T}$ – $G_{C}$ and $G_{T}$ – $G_{O}$, where $G_{T}$ is the energy of the transition state.

The allosteric coupling is realized by an additional contribution to the free energies of C and O, which depends on the state of the allosterically coupled channel. In the diagram, where the coupled channel is open, the additions result in the free energy curve in red. An amount of energy $\varepsilon_{CO}$, assumed positive, is added to the C state to produce the energy $G_{CO}$, and an amount $\varepsilon_{OO}$, this one negative, is added to the O state to yield $G_{OO}$. The modified equilibrium constant for opening of the closed channel will be

$$K_{O}=Ke^{-\left(\varepsilon_{OO}-\varepsilon_{CO}\right)}$$ (9)

where the subindex O refers to the state of the coupled channel. (Note that subindexes C that refer to C channels are in roman font, a convention that also applies to V channels, while those that refer to the “Closed” state are in italic font.) If the coupled channel were closed, the corresponding changes in energy would be $\varepsilon_{CC}$ and $\varepsilon_{OC}$, and the closing equilibrium constant would be

$$K_{C}=K^{-1}e^{-\left(\varepsilon_{CC}-\varepsilon_{OC}\right)}$$ (10)

Because C and V channels are assumed identical, $\varepsilon_{CO}$ and $\varepsilon_{OC}$ must be equal.

Diagram B also illustrates how the allosteric energies will change the kinetic rates. In the model, an additional parameter *ν* (also known as *η* or *δ*, “barrier effective electrical distance”) splits the energy change Δ between the two unidirectional rates, as *ν*Δ and (1 − *ν*)Δ. Thus, in diagram B, the energy difference Δ (which in the example is equal to the sum of the absolute values of $\varepsilon_{OO}$ and $\varepsilon_{OC}$) is used ∼25% to lower the forward, opening energy barrier and 75% to raise the closing energy barrier. As written in diagram C, the opening and closing transition rates are multiplied, respectively, by the exponentials of −*ν*Δ and −(1 − *ν*)Δ.

(Note that the energy profile modified by allostery can be shifted vertically, in the illustration to the curve in grey, without any effect on the calculated kinetics or equilibria. The shift in energies, which sets $\varepsilon_{CO}$ = $\varepsilon_{OC}$ = 0, is acceptable because the *G*’s and the interaction energies $\varepsilon_{OO}$, $\varepsilon_{CO}$, and $\varepsilon_{CC}$ are effectively unknowable; only their differences are measurable, via their effects on equilibria and transition rates, the sole effects that matter for the present simulation. In addition to simplifying Eqs. 9 and 10, the shift yields a better insight to the allosteric effects, visited later.)

In all, the properties of the allosteric contacts (of which there is only one sort, V-C or C-V) are defined by two parameters, $\varepsilon_{OO}$ and $\varepsilon_{CC}$, represented in kT units (where k is Boltzmann’s constant) plus the splitting coefficient v. Therefore

$$k_{CO\to OO}^{+}=k^{+}e^{-{v\varepsilon }_{oo}},k_{CO\to OO}^{-}=k^{-}e\left(1-v\right)\varepsilon_{OO}$$ (11)

$$k_{CO\to CC}^{+}=k^{+}e^{{-V\varepsilon }_{CC}}\;\text{and}\;k_{CO\to CC}^{-}=k^{-}e\left(1-v\right)\varepsilon_{CC}$$ (12)

A curious aspect of the V-C dichotomy, contemplated in the model and found to determine some of its unique properties, is that the V-C contacts are *multiple* and *exclusive*. Individual channels contact three others (*multiple*), all of them in the other class (*exclusive*), so there are no V-V or C-C contacts. Consequently, a C channel operation is not directly affected by other C channels; the old simile (Rios and Pizarro, 1988; Pape et al., 1995) of V channels as “masters” and C channels as “slaves,” which is heretofore replaced by “leaders” and “followers,” is upheld. However, the leader–follower relationship is promiscuous (interlaced might be a better term): every C channel has allegiance to three V leaders, while every V leader has three C followers, each with a different trio of leaders. It seems safe to expect that this intricate arrangement will bring extraordinary physiology.

Two more features must prevail: the interaction must be reciprocal; a closed C channel will be nudged to open by a neighbor open V channel if their $\varepsilon_{OO}$ is negative, while an open C channel will facilitate a neighbor’s V channel opening by the same $\varepsilon_{OO}$. The equality is trivial because channels C and V are assumed identical, but would still apply if they were not, to ensure microscopic reversibility (see diagram D in Fig. 9, in which identity of C and V is assumed, and Box 1, which develops the case for not identical channels). This reciprocity has interesting consequences: the state of every C channel is influenced by its three V neighbors and vice versa for every V channel; therefore, and intriguingly, the followers have sway on the behavior of their leaders. The interlocking of the leader–follower relationship, noted earlier, has the consequence that these interactions travel through the whole set of channels making the entire couplon an excitable continuum, which adds to both the challenges of the simulation and the richness of the final result.

Box 1

**Figure:**
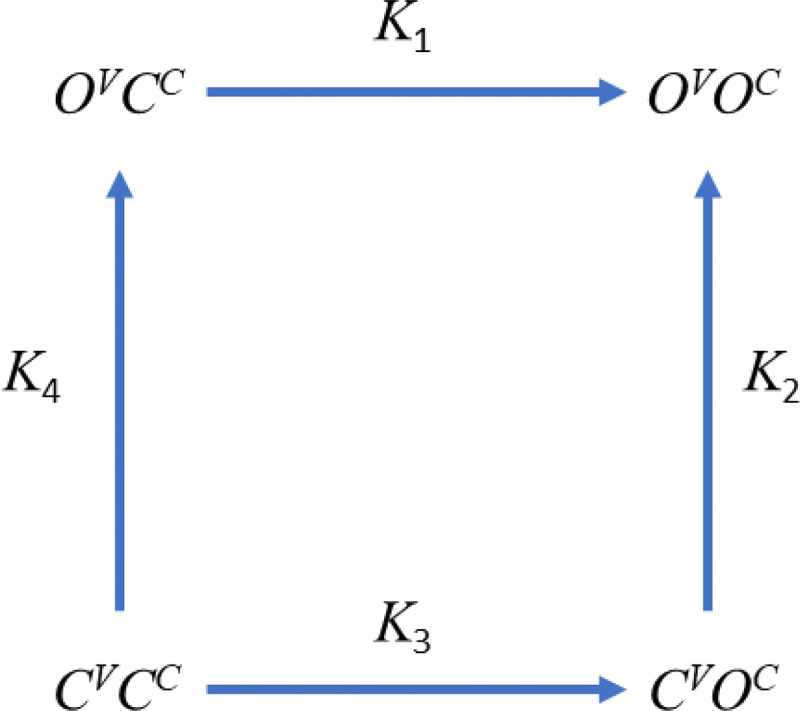

To demonstrate symmetry of allostery when channels V and C are not identical. The channels to which the state symbols and energies refer are now identified with roman superindexes. $K_{1}$ and $K_{3}$ refer to gating of the C channel, and $K_{2}$ and $K_{4}$ to the V channel.

With this differentiated formalism, $K_{1}$ is expanded as

$$K_{1}=\frac{k_{C}^{+}e^{-\nu^{C}\varepsilon_{OO}^{C}}}{k_{C}^{-}e^{-\left(1-\nu^{C}\right)\left(-\varepsilon_{OO}^{C}\right)}}=K_{C}e^{-\varepsilon_{OO}^{C}}$$

where $K_{C}$ is the equilibrium constant of channels C, no longer assumed identical to $K_{V}$. ($K_{C}$, with roman subindex, should not be confused with $K_{C}$, the closing equilibrium constant in Eq. 10). The other equilibrium constants can be similarly written as

$$K_{2}=K_{V}e^{-\varepsilon_{OO}^{V}}$$

$$K_{3}=K_{C}e^{\varepsilon_{CC}^{C}}$$

$$K_{4}=K_{V}e^{\varepsilon_{CC}^{V}}$$

The microscopic reversibility condition: $K_{1}K_{4}$ = $K_{2}K_{3}$, requires the equality

$$\varepsilon_{CC}^{V}-\varepsilon_{CC}^{C}=\varepsilon_{OO}^{C}-\varepsilon_{OO}^{V}$$

which could not be satisfied at all possible V, unless both sides are identically zero. The two identities restate the reciprocity of the allosteric effect even without the assumption of identity between C and V channels.

#### Activation by voltage

Activation is modeled as starting at V channels. It is implemented in the same way as allostery. An increment in membrane voltage is assumed to cause proportional changes in the free energies of both closed and open V channel states, so that opening is favored. Unlike allosteric interactions, voltage-dependent activation of channel opening is assumed to be an irreversible process that requires an injection of energy, as the return of channels to the resting state in the model generally takes a different route to that of activation, dissipating energy in the cycle.

The energy source is electrical, applied upon membrane depolarization as *work* on four Ca_V_s for every V channel. The maximum energy that can be passed to the RyR is that provided by a change Δ*V* to the moving charge *ze* coupled to gating. *ze* can be estimated under three assumptions: (1) all four Ca_V_s engaged by one RyR1 must move to cause it to open, (2) only one of the four VSDs in Ca_V_1.1 is involved, and (3) two charged residues in the moving S4 helix must traverse the membrane electric field in the process. Assumption (1) may lead to a ∼25% overestimate of the energy available, as work in progress by G. Brum and Rios favors three sensors, instead of four, as a minimum of the number of Ca_V_s required. Assumptions (2) and (3) are informed by work of Riccardo Olcese’s (Pantazis et al., 2014; Angelini et al., 2025) and Bernhard Flucher’s groups (Pelizzari et al., 2024; Heiss et al., 2025). Under these assumptions, if 4 (Ca_V_s) × 1 (domain) × 2 elementary charges undergo a 100-mV change, the energy input will be 0.8 eV or 30 kT. The present model requires the transfer of less than half of that amount to maximally open a V channel.

Channels V transition between two states according to the state diagram Eq. 8. Because there is no reason to do otherwise, in the resting state all channels, V or C, are assumed to have the same transition rates. They are set at $k^{+}$ = 0.001 and $k^{-}$ = 2, in ms^−1^, to make the closed state overwhelmingly dominant (K = 2,000^−1^). V channels are driven to open by the electric field, assumed to add an amount $\varepsilon_{V}$ (in kT units) to the energy difference between ending (O) and starting (C) states. $\varepsilon_{V}$, a negative number, is assumed proportional to V (as it is derived from the electric work done on the sensors), ranging from 0 at the resting V to −14 at maximal activation. As just discussed, the electrical work done on the subset of voltage sensors involved is amply sufficient to satisfy this energy requirement.

(A note on signs: while the energy contribution by membrane depolarization, named $\varepsilon_{V}$, enters as a negative term of Δ*G* in the opening direction, the electric work is applied on the RyR for a gain—positive. There is no contradiction, as the work is applied on the closed channel and Δ*G* is, conventionally, *G*_open_ – *G*_closed_.)

An approximate equivalence in volts of these energies emerges from taking −90 mV as the resting potential (corresponding to $\varepsilon_{V}$ = 0) and noting that a pulse to +10 mV saturates release flux (Ferreira Gregorio et al., 2017), corresponding to $\varepsilon_{V}$ = −14 kT; therefore, −1 kT is delivered by a depolarization of 7.14 mV.

By analogy with Eq. 9, the electric energy input will modify the equilibrium constant for opening of V channels as

$$K_{V}=Ke^{-\varepsilon_{V}}$$ (13)

and the unidirectional transition rates as

$$k_{V}^{+}=k^{+}e^{-v_{V}\varepsilon }\text{ and }k_{V}^{-}=k^{-}e\left(1-v_{V}\right)\varepsilon_{V}$$ (14)

A subindex V is used in Eqs. 13 and 14 to mark the parameters that apply to V channels.

In addition to the electrical energy contributed by voltage sensors, channels V receive allosteric energy contributions from linked C channels ($\varepsilon_{\text{OO}}$ and $\varepsilon_{\text{CC}}$), according to the rules established in the previous section.

#### Inactivation

**Figure 10.**
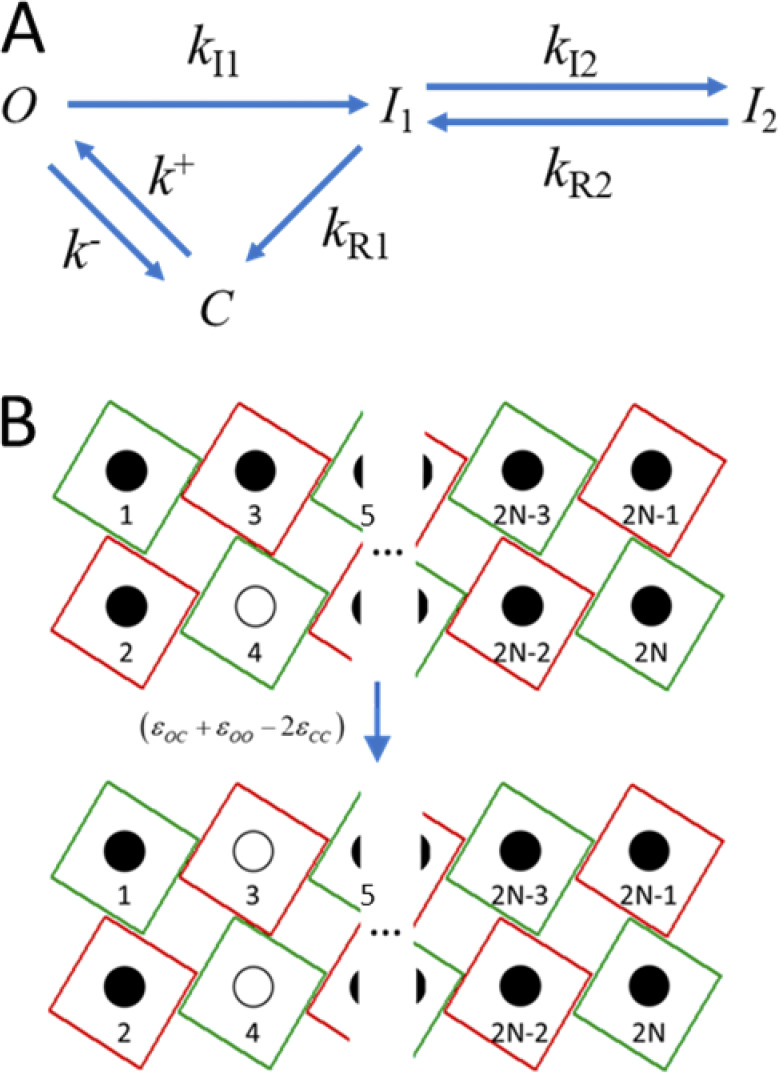
**Channel states and allosteric interactions. (A)** State diagram for C channels. O: open; C: closed, $I_{1}$ and $I_{2}$: inactivated. V channels only have O and C states. Note irreversibility of $O\to I_{1}$ and $I_{1}\to C$ transitions. **(B)** Illustrates the allosteric energy change in the opening transition of channel 3, when one of its three linked channels (#4) is open and the others (#1 and #5) are closed. Upon opening, all three interactions of #3 change: two CC are lost (with #1 and #5), replaced by two OC, and gains one OO (with #4), replacing one OC, for the net energy change listed. **(B)** Also illustrates crucial features of the model couplon. The two categories, V and C, are represented in different colors (which one is V is not important here). Note that in large couplons every channel except two at each end interacts with three others, all of which are in the “other” category (every red one with three green ones, and vice versa). In 4-channel couplons every channel interacts just with two others. An *N* value of 30 (i.e., 30 V and 30 C channels) was used for the simulations of macroscopic or cell-level flux. Simulations for comparison with bilayer data were done with totals of four channels (*N* = 2) or six channels (*N* = 3).

Inactivation (defined as passage to a closed state incapable of activation) determines the decay from the peak of release flux in the continued presence of depolarization. A vast literature, starting from Schneider and Simon (1988), indicates that the increase in [Ca^2+^] that the channels face on their cytosolic side upon Ca^2+^ release is involved in its causation, but the actual mechanism is poorly defined. Based on experiments such as the one in Fig. 8, we do not include in the model any explicit effect of [Ca^2+^] and assume instead that inactivation is linked to activation by simple rules described with Fig. 10 A.

According to this diagram (which, as discussed, only applies to model channels C because channels V are assumed not to inactivate), channels are in an open–close (O↔C) equilibrium defined by rates $k^{+}$ and $k^{-}$, which is modified allosterically by the coupled V channels according to Eqs. 11 and 12. Open C channels may inactivate to $I_{1}$, where they are closed—that is, they do not pass flux and they interact allosterically as closed channels. From $I_{1}$, channels can recover to C or go into a second inactivated state $I_{2}$, from where they can only transition to $I_{1}$. These rules make inactivation nearly “fatal” or “deterministic,” because, in simulations with optimized parameter values, most channels that open during a pulse end up in an inactivated state.

(The case for the connections between O, C, and $I_{1}$ shown in Fig. 10 was made in detail by Pizarro et al. [1997], based on observations in *Rana*, and in the mouse by Szentesi et al. [2000]. Briefly, if the suppression of a maximal release by a small conditioning transient is equal to the decay during conditioning, it implies that only the channels that opened in the conditioning transient became inactivated—demonstrating C←I unidirectionality. Additionally, that a conditioning pulse greater than the test pulse cannot increase suppression beyond that induced by one of equal amplitude implies that every channel susceptible to inactivation during the test, i.e., those that opened, did so—otherwise they would have inactivated when preceded by the larger conditioning. Which means that every channel that opens inactivates, requiring O→I unidirectionality.)

That the $O\to I_{1}$ and $I_{1}\to C$ transitions are assumed irreversible implies that channels cycle around this ring, cycling that will be fast at high levels of activation. That the scheme works without taking Ca^2+^ explicitly into account tells that the ion’s inhibitory action is contained in these rules. A mechanism whereby these effects could be achieved without an explicit consideration of [Ca^2+^]_cytosol_ is described in Discussion.

The need for a second, “deep” inactivated state, $I_{2}$, is demonstrated in the first section of Appendix C, showing that simulations without it can be made to match some but not all of the observed properties; for example, slowing recovery from $I_{1}$ to match the observed time course of the restoration of flux leads to an erroneous voltage dependence of the ratio Peak/Steady flux, etc.

## Materials and methods

The Ca^2+^ flux emitted by a single couplon with structure diagrammed in Fig. 10 B: 30 V and 30 C channels (respectively, green and red squares) in checkerboard double row, contacting Ca_V_s in the skipping pattern illustrated with Fig. 1, was computed numerically.

The simulation starts from a resting configuration, consistent with the state in a well-polarized cell, at a potential defined as −90 mV. Channels V transition between two states according to the diagram Eq. 8. In the resting state, all channels, V or C, have the same kinetic rates ($k^{+}$ = 0.001 ms^−1^, $k^{-}$ = 2 ms^−1^), being effectively closed. V channels are driven to opening by depolarization, which changes the equilibrium constant and transition rates according to Eqs. 13 and 14. Under the assumptions, the equilibrium constant goes from 0.0005 at rest, to 1 at ∼7.6 kT*,* the midpoint, and to ∼4,500 at −14 kT, the saturating value of $\varepsilon_{V}$.

Opening of V channels biases C channels to open by allostery. Fig. 10 B illustrates the allosteric influences experienced by channel #3 in the opening transition; the effects of its three linked V channels must be taken into account. As detailed in the figure legend, the net energy change is $\varepsilon_{OC}+\varepsilon_{OO}-2\varepsilon_{CC}$, which simplifies to $\varepsilon_{OO}-2\varepsilon_{CC}$ as ε/OC = 0. With the parameter values that best simulated the observations, $\varepsilon_{OO}=-5.7$ and $\varepsilon_{CC}=-0.7$, the opening transition in channel #3 will be amply favored.

Reciprocally, when a V channel transitions, the connected C channels contribute allosterically to the energy change using the same rules and the same parameter values (see examples in Fig. 10).

Unlike V channels, channels C inactivate according to state diagram A in Fig. 10. The rates for inactivation and recovery are assumed constant, i.e., independent of voltage and allosteric effects (assumptions justified only for their simplicity, as more parameters would be needed to describe alternatives for which there is no evidence).

With these definitions and assumptions, the couplon is a memoryless cluster of channels (the evolution of which only depends on the present state and the present conditions, namely the voltage V and nothing else). The evolution of the array over time is calculated as a 2N-valued stochastic Markov chain ***S***(*t*), a succession of vectors (sets) ***S*** of state values (one value, C or O, per V channel; those two plus $I_{1}$ and $I_{2}$ for C channels) determined by rules that depend only on the present set of channel states and V. The simulations consist of building the Markov chain, i.e., the succession of state values for every channel in the couplon, which is done following Gillespie (1976) with operational details described by Groff and Smith (2008). A Markov “run” (i.e., a realization of the chain ***S***(*t*)) has as main output the functions *n*_C_(*t*) and *n*_V_(*t*) from which flux is calculated by Eq. 7. The simulated fluxes compared with observations below are averages of between 800 and 6,400 Markov runs. Individual runs are presented in the last section of Results.

The Open ←→ Close evolution of all channels in the set is computed via stochastic decisions that start by calculating transition rates for every channel to then decide what channel makes the “next” transition. The decision is reached via lottery—a random variable uniformly distributed between 0 and 1 is applied to a segment of length one partitioned among the 2N channels according to their calculated rates. Kinetics is introduced by timing each transition by the collection of calculated rates; thus, the *expected* time of each transition is computed as the inverse of the sum of the individual rates over all channels in the couplon. Once this individual transition—called “event”—is decided (once its transitioning channel is identified), the time of occurrence is associated to the event; thus, ***S***(*t*) is built as a sequence of configurations at unequally spaced time points *t*_event i_. If event *i* is a channel opening, two procedures follow: one repeats the procedure done before, to decide on the next O←→C transition (event *i* + 1); the other consists in checking for every C channel, open or inactivated, whether it should undergo any of the inactivating and recovering transitions available to it (closed channels are excluded, as they cannot inactivate).

The inactivating/recovering decisions are also reached stochastically, as follows: every time an event occurs and its locus (channel *n*) and time (*t*_event i_) are recorded, an “alarm” is set that will define the next transition of channel *n*. Thus, if C channel *n* transitioned to O, the time at which this happened (*t*_n,open_) is recorded (the “clock” starts ticking), and the alarm is set by a lottery that defines its dwell time before inactivation, *dwell*_n,I1_, generated as a random number with exponentially decaying distribution of rate constant *k*_I1_ (In actual implementation, it is more practical to use the parameter “inactivation 1 half-life,” *I*_1_*_ht*, equal to log2/*k*_I1_). When a new event occurs (event *i* + 1, a closing or opening of any channel, V or C, decided stochastically), the interval elapsed between the last time a C channel opened and the new time point (*t*_event i+1_ - *t*_n,open_) is compared with the respective *dwell*_n,I1_; if the difference is greater than the dwell time, the state of channel *n* is changed to *I*_1_. This is done for every open C channel. If instead channel *n* enters state *I*_1_, the time (*t*_n,I1_) is recorded and two alarms are set; two exponentially distributed random dwell times: *dwell*_n,R1_, of rate constant *k*_R1_ (time to recovery), and *dwell*_n,I2_, of rate constant *k*_I2_ (time to transition to *I*_2_). The faster transition takes precedence; upon event *i* + 1, the elapsed time (*t*_event i+1_ - *t*_n,I1_) is compared with the smallest of the two dwell times; if the elapsed time is longer, channel *n* undergoes the transition to the corresponding state (Because these dwell times are random, a channel will not always follow the path of greatest transition rate). Finally, the sojourn of a channel at *I*_2_ will be determined as for *I*_1_ but by a single random variable, *dwell*_n,R2_.

The optimized adjustable parameter values are listed in Table 1. As argued in Discussion, the parameter space is highly constrained, both by couplon structure and because the parameter values are highly correlated.

**Table 1.** Model parameter values applied throughout, except in the simulations of 4- and 6-channel couplons, for which the transition rates were changed to 0.125 and 0.125 ms^−1^

Parameter	Value	Dimension, unit	Description
$k^{+}$	0.001	Time^−1^, ms^−1^	Opening transition rate
k	2.0	Time^−1^, ms^−1^	Closing transition rate
v	1.0	None	Allosteric distribution
$v_{V}$	0.8	None	Electrical distribution
$\varepsilon_{OO}$	−5.7	Energy, kT	Allosteric energy, open
$\varepsilon_{CC}$	−0.7	Energy, kT	Allosteric energy, close
${I_{1\_}}_{ht}$	3.5	time^−1^, ms^−1^	Inactivation half time
${I_{2\_}}_{ht}$	50	time^−1^, ms^−1^	Second inactivation half time
${R_{1\_}}_{ht}$	20	time^−1^, ms^−1^	Recovery half time
${R_{2\_}}_{ht}$	50	time^−1^, ms^−1^	Second recovery half time
$R_{C/V}$	5	None	Channel flux ratio C/V
$E_{S}$	−14	Energy, kT	Saturation energy

### Simulation errors and statistics

**Figure 11.**
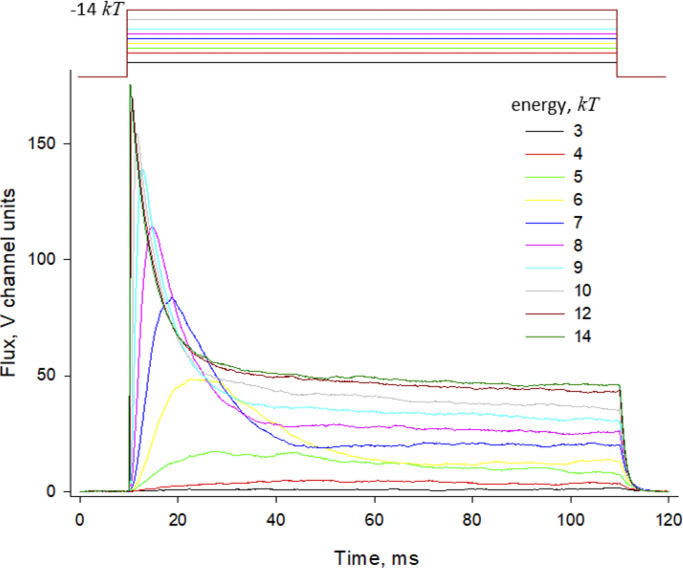
**Ca** ^**2+**^ **release flux elicited by constant energy pulses, color-coded in the top diagram.** This is the model output to be compared with experimental records of depletion-corrected flux (as in Figs. 4 B, 6 B, or 8 C). Each record is an average of 800 Markov chain realizations. The voltage changes corresponding to each pulse energy are specified in the text. The model parameters used for calculating these and all other averages are always the same, listed in Table 1. Flux, derived from the numbers of V and C channels open via Eq. 7, is given in units of the flux through an open V channel.

**Figure 12.**
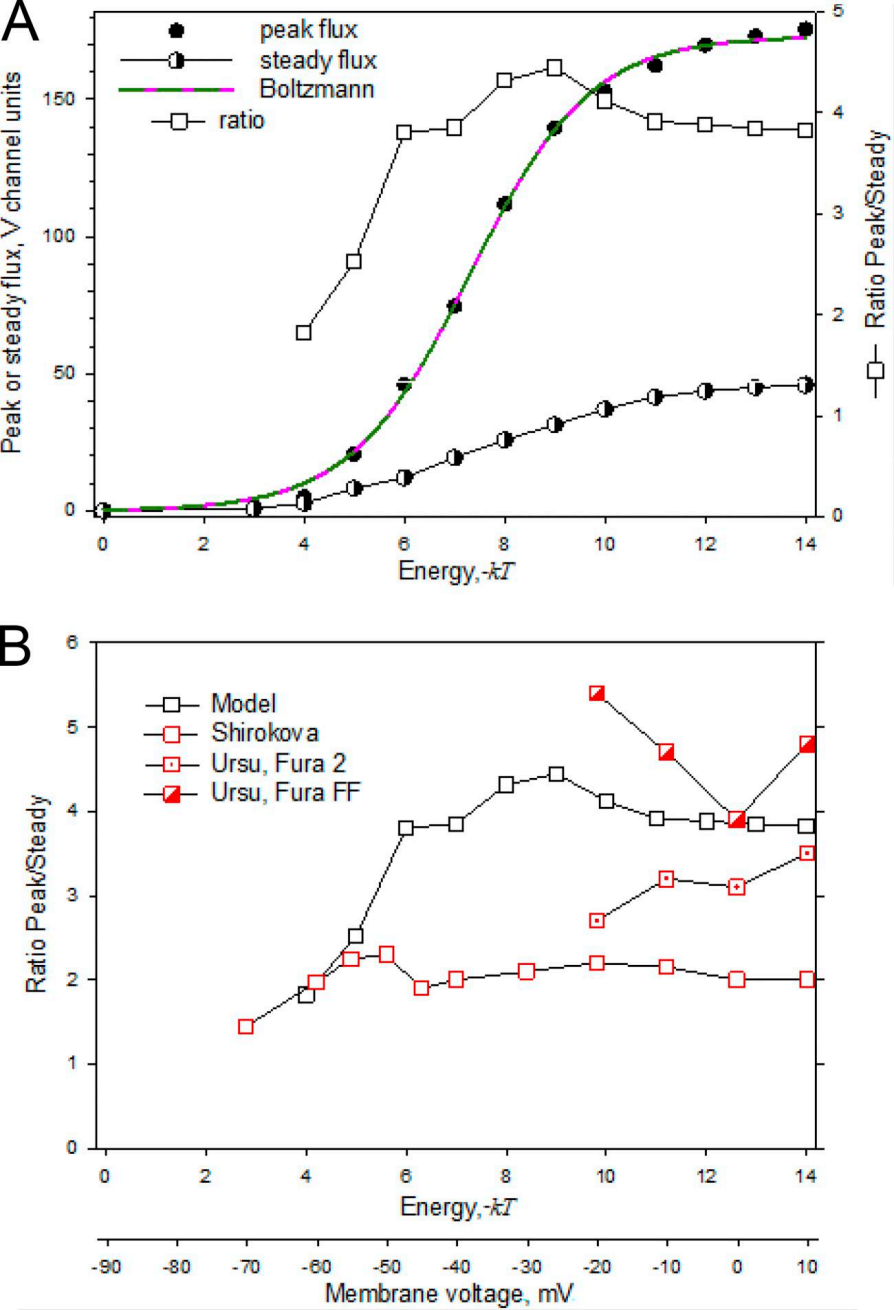
**Voltage dependence of simulated flux. (A and B)** Peak and steady values of flux and their ratios, vs. activation energy (equivalent voltages given by the second horizontal axis in B). The Boltzmann fit to the peaks (Eq. 15) yields $\bar{V}$ = −37.7 mV and κ = 8.5 mV). B, ratios (Peak/Steady) from panel A, compared with measurements by Shirokova et al. (1996) and Ursu et al. (2005) replotted from Fig. 4, D and H.

**Figure 13.**
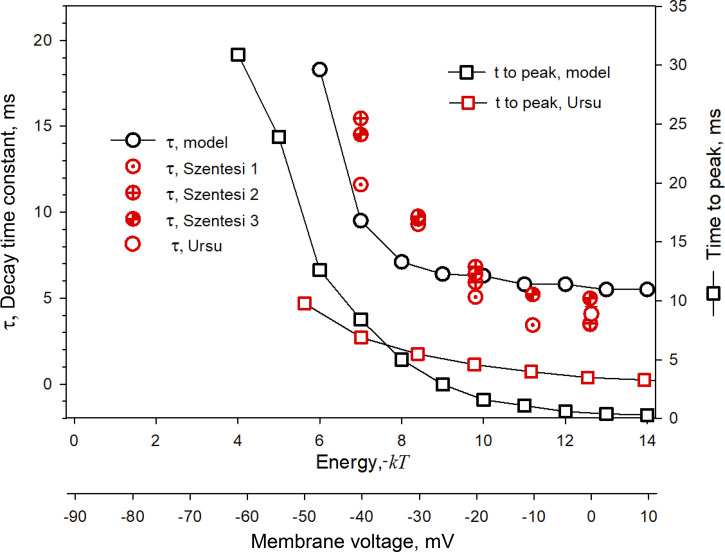
**Kinetics of simulated flux.** Times to peak and time constants of decay of the records in Fig. 11, black symbols, compared with experimental values in red. Times to peak from Ursu et al. (2005) and time constants from Szentesi et al. (2000) for “Szentesi 1,” Szentesi et al. (2004) for “Szentesi 2” and Ursu et al. (2005). The data from Ursu et al. (2005) were shifted by −30 mV in the voltage axis to reconcile with the voltage dependence found by Ferreira Gregorio et al. (2017), used here to establish the correspondence between energy and voltage.

**Figure 14.**
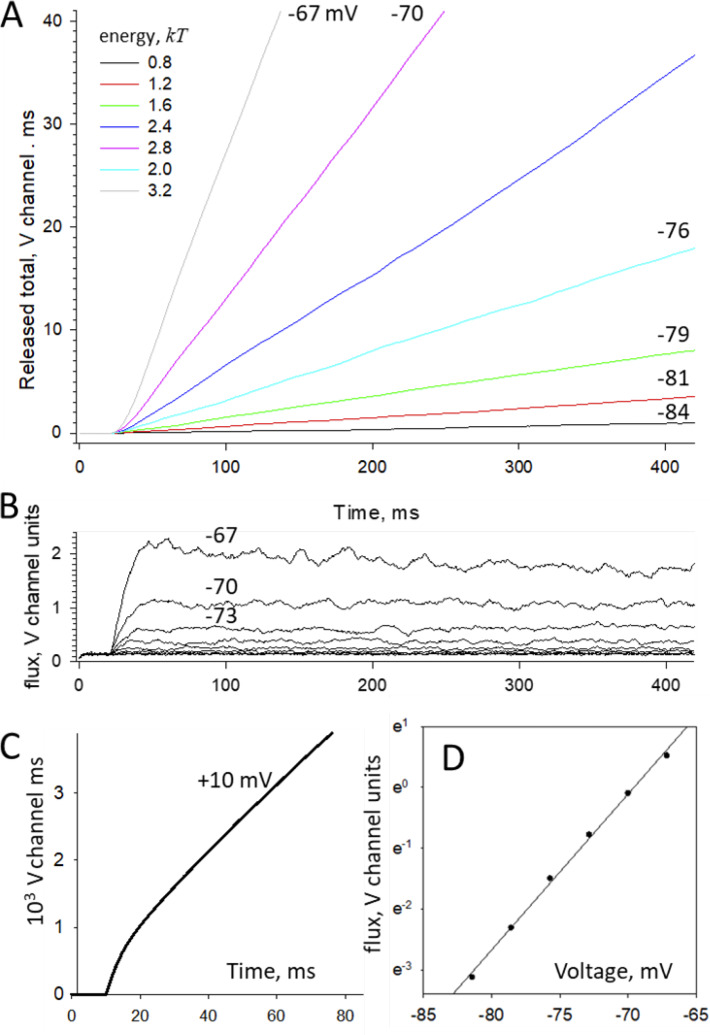
**Ca** ^**2+**^ **release flux at very low voltages. (A)** Time integrals of the simulated release flux at the voltages indicated next to each record, color-coded to energies applied (in legend). Integrals are shown for comparison with the experimental traces shown in Fig. 5 A. **(B)** The actual Ca^2+^ release fluxes, which are nearly constant—as they are the time derivatives of the traces in A. Records are averages of 6,400 Markov realizations for a 60-channel couplon. **(C)** An example time integral of flux at a high voltage, showing, by contrast with the records in A, a prominent maximum in the slope, corresponding to the early peak of the derivative (Ca^2+^ flux). **(D)** Semilogarithmic plot of flux vs. voltage, with linear fit corresponding to an *e*-fold increase in 3.91 mV, similar to the experimental value (Fig. 5 B).

**Figure 15.**
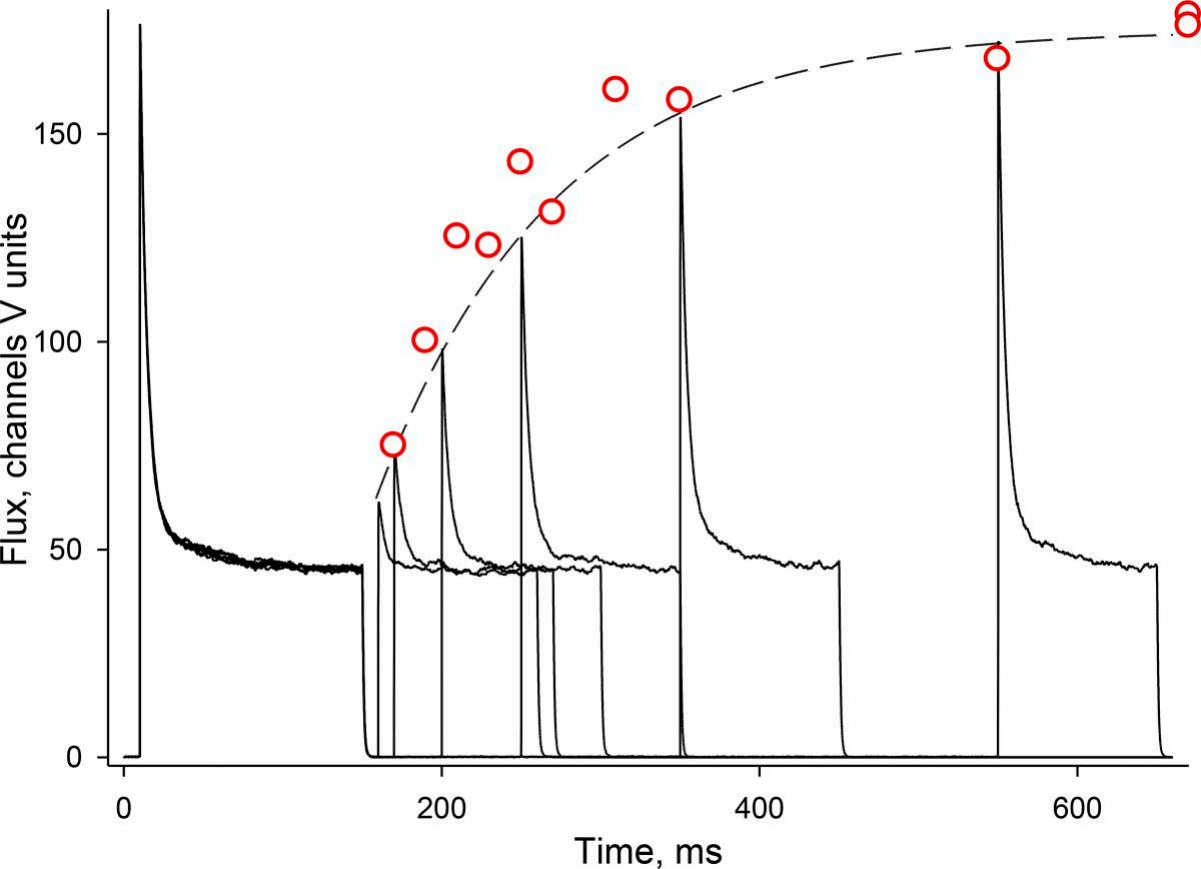
**Recovery from inactivation.** A pulse of 140 ms and −14 kT (corresponding to +10 mV) precedes a test pulse of the same amplitude and 100 ms duration, at different intervals between the end of the conditioning and the beginning of the test. The dashed line represents the best fit to the test peak flux $F_{P}$ (interval) function, FP=FS+(FP,∞−FS)(1−e−intervalτ) with τ = 110 ms. The red circles are values measured by Sárközi et al. (2000), plotted in their Fig. 7B and kindly shared by Péter Szentesi. τ in this case was 92.1 ms. (the two superimposed datapoints at the end of the plot correspond to interval values outside the plotted range).

**Figure 16.**
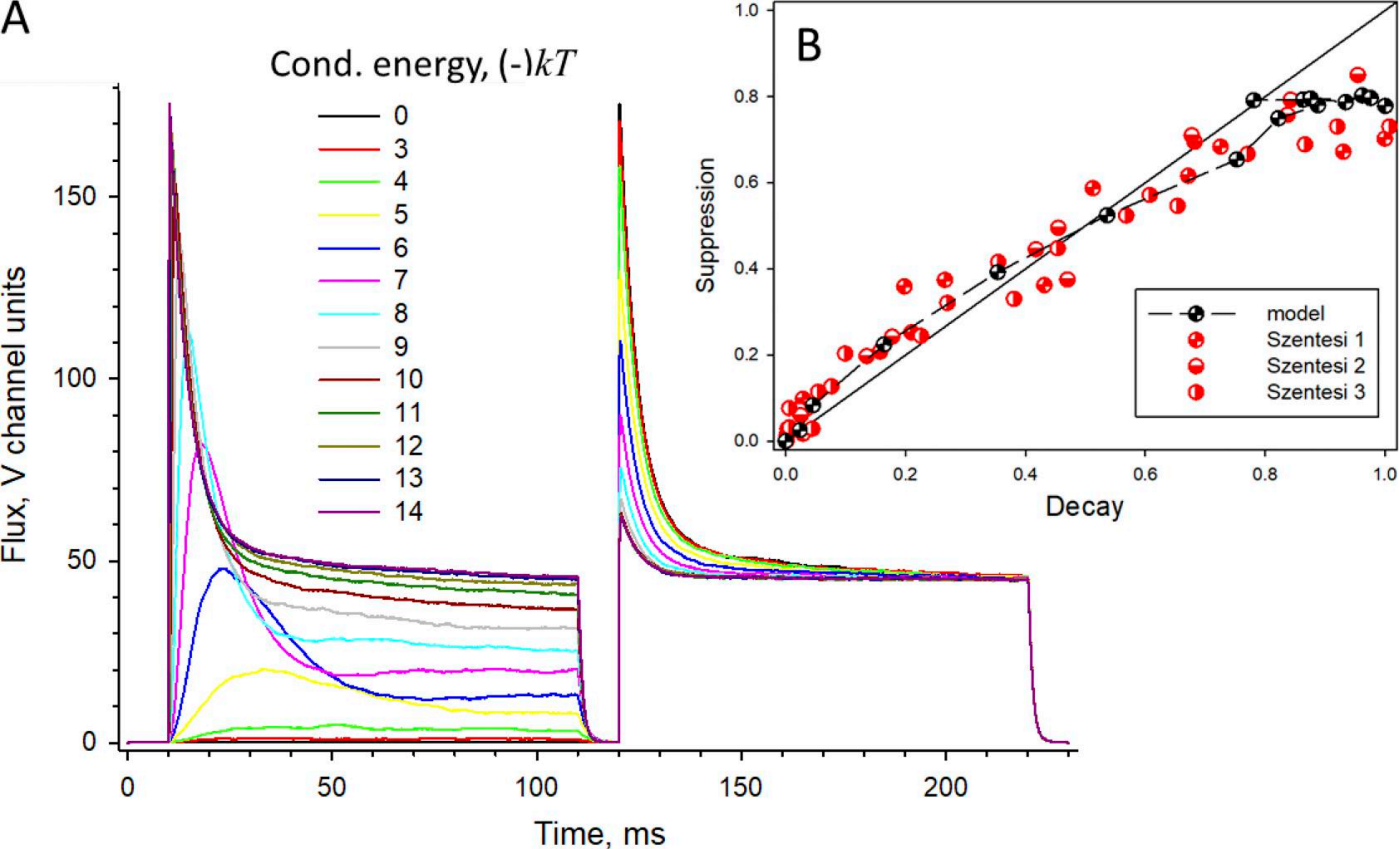
**Near-equality of decay and suppression. (A)** Simulation of the flux elicited by a conditioning-test pair of pulses. The test energy/voltage is high (−14 kT, corresponding to +10 mV) and the conditioning energy varies, spanning the range [0 to −14 kT] as listed. **(B)** Black symbols: suppression in the test pulse vs. decay in the conditioning (defined with Fig. 6). Red symbols, “Szentesi 1”—data measured on rat muscle by Szentesi et al. (2000). “Szentesi 2” and “Szentesi 3” data in Szentesi et al. (2004), kindly provided by Péter Szentesi.

**Figure 17.**
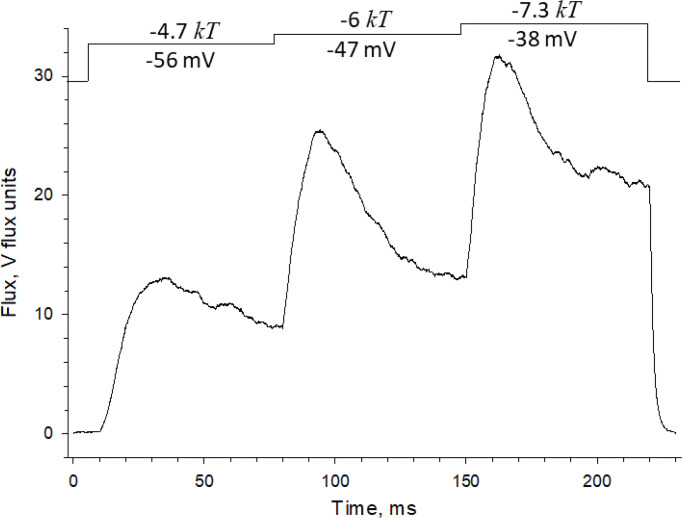
**The model features increment detection.** Simulated flux for a couplon activated by the three-step voltage pattern shown at top. Average of 2,400 realizations. For comparison with increment detection in frog muscle (Fig. 7).

**Figure 18.**
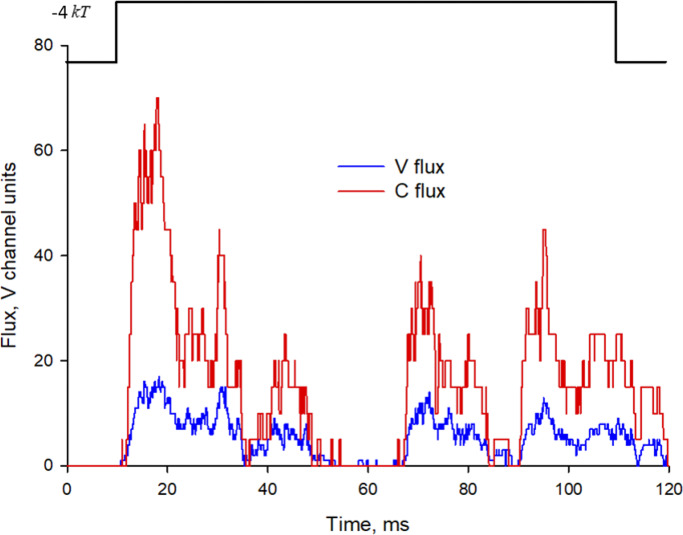
**Simulation of a local event.** Flux generated by a single run of the Markov chain of the 30 channel couplon, elicited by a −4 kT pulse (equivalent to −54 mV). Parameter values are the same as for the previous records, listed in Table 1. Fluxes through V and C channels are plotted separately. The unit in both cases is the flux through one open V channel. Both variables take discrete values, with steps of value one in the V channel case, and five (i.e., RCV) for C channel flux. Note that channels tend to open in sets, rather than individually. Note also that decay of the C channel current after a local peak during the pulse is usually accompanied by V channel closure—a consequence of the tight reciprocal allosteric coupling. Multiple Markov realizations in the same conditions are plotted in Supplement 2, Fig. S2 1.

All simulations presented are derived from realizations of the Markov chain ***S***(*t*) representing the sequence of flux values upon stimulation of a single couplon. Individual realizations are illustrated at the end of Results. All other traces (in Figs. 11, 12, 13, 14, 15, 16, 17, and 18) are of averages of individual “runs,” 800 in most cases, 6,400 for the responses to very low voltages (Fig. 14). The inherent error of the averages is first analyzed point-by-point, that is, at every time point in the simulation. From this time point–dependent error, a collective error of the average over realizations is derived at every point. In turn, the time point–dependent error of the average is the basis to calculate errors in emergent measures (for instance, steady value of flux or time constant of decay).

Through an analysis presented in Appendix A, the time point–dependent standard deviation of the individual Markov realization was estimated as 10 units of V channel flux during high-voltage pulses (the appendix shows the error’s variation with voltage). The time point–dependent standard deviation of the average of 800 realizations is therefore 10 × 800^−0.5^ or 0.35. This deviation, reduced further in the emergent measures, is small as a fraction of mean fluxes of tens of units; therefore, no more replications seem necessary. For the very low fluxes elicited at low voltages (Fig. 14 B), of the order of 1 or 2 units, 6,400 realizations were averaged, with consequent reduction of the standard deviation to 0.15. The deviation estimates, 0.35 and 0.15, are consistent with the visual appearance of the average records, respectively, in Fig. 11 and Fig. 14 B.

### Coding, equipment, and computing load

The description of methods is completed with Supplement 3, where the computational code that implements the model is shared, and Appendix B, the preprocessing and code of a simple application that digitizes a graphic plot (both in IDL language, NV5 Geospatial, Paris, France). To ease the use of these techniques, we share in Supplement 5 a transcription of the digitization program to Python, made by the AI assistant Claude (by Anthropic) and checked by us for functionality.

All calculations were performed on a well-equipped desktop computer (Dell-brand, “Precision” 3630 Tower model, with an Intel Core i7-9700K CPU processor—8 cores, 8 threads—clocked at 3.6 GHz, with 16 GB RAM).

Processing time for a flux simulation at one voltage depends linearly on the number of channels in the couplon, the number of time points (= pulse(s) duration/time resolution), and the number of averaged repetitions. They also depend on the energy of the pulse (as higher levels induce more transitions per unit time). A typical simulation of flux through a 60-channel couplon as shown in Fig. 16, of 230 ms at 20 µs resolution (i.e., 11,500 time points), at mid-voltage, with 1,000 repetitions, took ∼30 min of computing per pulse pair. The full simulation represented in Fig. 14 took ∼28 h.

### Appendices and online supplemental material

Appendix A contains a determination of simulation errors. Appendix B discussed a procedure to extract digital records from analog plots. Appendix C contains alternative versions of the model. Data S1 includes supplemental text and images at the end of the PDF document, which provide information about simulations of flux in smaller couplons (Supplement 1), individual Markov runs in large couplons (Supplement 2), simulations with depolarizing pulses of finite risetime (Supplement 3), annotated code of the basic simulation program (Supplement 4), and Python code of the digitization program (Supplement 5).

## Results

The results are presented in three sections, at the whole-cell, local couplon, and single-channel levels. The whole-cell fluxes are simulated as averages of realizations from a single couplon with 60 channels (additional results for smaller couplons are in Supplement 1). Single realizations of a large couplon correspond to the experimental observations at couplon level (Fig. 3). Finally, the single realizations of a 4- and a 6-channel array are compared with the bilayer currents of coupled channels of Porta et al. (2012) shown in Fig. 2.

### The simulations have adequate voltage dependence and kinetics

#### Voltage dependence

The set of fluxes elicited by constant voltage pulses of 100-ms duration is displayed in Fig. 11. The waveforms feature the early peak decaying to a steady level seen in the experimental records (as in Fig. 4, A and B). The peak and steady flux values grow monotonically with the electrical energy, which is added in quanta of −1 kT. The dependence, displayed in Fig. 12 A is sigmoidal, saturating at −14 kT (energies are always less than or equal 0; the minus sign is systematically omitted in the plots). The dashed curve plots the Boltzmann fit to the peaks

$$F_{P}=F_{\max}{\left(1+e^{-\left(\varepsilon_{V}-{\bar{\varepsilon }}_{V}\right)/\kappa }\right)}^{-1}$$ (15)

with fit parameters *F*_max_ = 173, ${\bar{\varepsilon }}_{V}$ = −7.32 kT, and κ = 1.19 kT. As discussed in Theory, the Boltzmann parameters can be converted to electrical units. Namely, −1 kT corresponds to +7.14 mV; therefore $\bar{V}$ = −90 mV - 7.32 kT × (−7.14 mV/kT) = −37.7 mV; likewise, the slope factor k corresponds to 8.5 mV. The reference experimental parameter values in the fit by Ferreira Gregorio et al. (2017) were $\bar{V}$ = −39 mV and κ = 8.9 mV. Likewise, *V* of half effect was −36 mV in the experiments of Shirokova et al. (1996). The model therefore achieves a good fit of voltage dependence.

(Note, however, that while the values of κ are consistent throughout the literature, those of $\bar{V}$ vary over a wide range, probably a result of their dependence on the ionic composition of the experimental solutions and an intrinsic variability in their primary readiness to open, manifest in the “transfer function” (Ríos et al., 1993) linking voltage sensor movement to RyR channel opening.)

Simulations with smaller couplons yield flux records (illustrated in Supplement 1) with minor quantitative differences. There is a systematic shift of $\bar{V}$ to higher depolarization as the couplon size is reduced. Thus, for a couplon of 30 channels, $\bar{V}$ = −35.2 mV, and for 14 channels $\bar{V}$ = −31.3 mV. Also, the effective valence decreases slightly (κ = 8.9 mV for the 30-channel couplon and 9.85 mV for 14 channels). Smaller couplons are therefore somewhat less reactive to voltage. This sensitivity could also contribute to the variability in the voltage dependence of activation of flux observed experimentally.

#### F_P_/F_S_ flux ratio

The ratio between peak and steady flux values is seen as highly relevant to mechanism since Shirokova et al. (1996) demonstrated that in *Rana* the ratio exhibits a prominent mode at intermediate voltages, while in mammals it is either flat (Shirokova et al., 1996) or rising steadily over the voltage range (Ursu et al., 2005). The differences are attributed to the contribution by CICR to Ca^2+^ release in amphibians. Ratios in the present simulation are compared with the experimental measurements by Shirokova et al. (1996) and Ursu et al. (2005) in Fig. 12 B. The parameter $R_{C/V}$ was set at five, largely to match the high temporal resolution measurements by Ursu et al. (2025). $F_{P}/F_{S}$ rises in the low voltage range in both observations and simulation, as the peak of flux develops in this range. In the simulations, it corresponds to a range where individual Markov runs rise less and with variable lags after the start of depolarization. In other words, while low voltages fail to synchronize channel opening, intermediate and high voltages fully engage the reactivity of the couplon. The relatively flat voltage dependence of the ratio at higher voltages proved independent of the value chosen for $R_{C/V}$, which instead determines the level at which the ratio stabilizes. As with the voltage dependence of peak flux, that of $F_{P}/F_{S}$ is similar across a range of model couplon sizes (Supplement 1).

#### Kinetics

The kinetic properties of the simulated waveforms are illustrated in Fig. 13. Exponential time courses of decay were fitted to the flux records starting from the time at which flux had decayed to 0.9 of peak level. As was the case for the experimental records, the time constant decreased monotonically with voltage. The values were close to those measured experimentally by various laboratories (in red).

Times to peak are also compared in the figure. The model values (black) tended to 0.5 ms at high voltage, while the fastest measurements of Ursu et al. (2005) approached 2–3 ms. That the mismatch is in part due to the use of voltage pulses of instantaneous rise is demonstrated by substituting pulses of finite risetime in Supplement 3. The issue will be addressed further with other concerns in Discussion.

#### Flux at very low voltages

A notable feature of the observations is the complete absence of a peak in the flux waveforms at very low voltage, which instead rise monotonically to a sustained level. This feature, only quantified in detail in frog muscle, is illustrated with Fig. 5. The comparable simulation is in Fig. 14. Because the flux at these voltages is very small, Pape et al. (1995) only showed its integral over time. The integral of flux in the simulation, in Fig. 15 A, yields a similar result: rises of nearly constant slope. The actual flux at every voltage is graphed in Fig. 15 B, demonstrating near constancy for all but the highest voltage in this low-voltage set. Fig. 15 C represents the integral of the flux simulated at a much higher voltage to show the early curving corresponding to the flux peak.

Panel D in Fig. 14 plots flux, averaged over the pulse duration, vs. V. As in the experimental observations of Pape et al. the dependence is exponential. Remarkably, the single parameter of the function, voltage increase for *e*-fold change, 3.91 mV in the simulation, is very similar to the experimental value reported by Pape et al. (3.73 mV). The near agreement of this parameter, a measure of the effective valence of the voltage sensor, is remarkable because it was not sought. Indeed, we set the applied electrical energy so that channel gating saturates by +10 mV, with no concern for the sensor valence required to extract the necessary energy. The implications of this agreement are considered in Discussion.

### Conditioning and inactivation are simulated well

#### Recovery from inactivation

The next simulations targeted the effect of a conditioning pulse on the waveform elicited by a subsequent test. A large enough conditioning essentially eliminates the peak of an ensuing test waveform placed close enough (as shown, for instance, with Fig. 6 in mouse muscle and Fig. 8 in the frog). In Fig. 15, illustrating time-dependent recovery from inactivation, flux is elicited by two maximal depolarizations at increasing intervals. Recovery is shown to follow an approximately exponential function of time, with a time constant of 110 ms. The individual circles are experimental values in the study of Sárközi et al. (2000), shared by the authors, which were fitted with a time constant of 92 ms; these values are representative of a set of replications by Sárközi et al. (2000), with time constants averaging 115 ms (SEM = 15 mV). The present simulations match their detailed set of measurements.

#### Quantal release and deterministic inactivation

The model output is compared next to the quantal aspects of inactivation. The reference study in mice by Szentesi et al. (2000), illustrated with Fig. 6, found near-equality of *decay* in flux by conditioning pulses of variable voltage and *suppression* in flux elicited by a large test voltage. The flux simulation with conditioning pulses spanning the range of energies between 0 (no conditioning) and −14 kT on the flux elicited by a maximal (−14 kT) test pulse is represented in Fig. 16 A. The values of decay and suppression are cross plotted in Fig. 16 B (black circles). The red symbols replot the experimental values from Fig. 6 C and an additional publication by the Debrecen group (Szentesi et al., 2004), with suppression and decay values normalized in every case to decay in the maximal conditioning flux.

In addition to the general agreement with experimental data, it is notable that simulation and experimental values deviate similarly from full equality between suppression and decay (a systematic excess of suppression in the low-to-mid range, −4 to −6 kT, and a marked deficit at the high end of the range). In mechanistic terms, the deviation at low voltages can be described as inactivation that “escapes,” invading channels that had not been activated during the conditioning pulse. The excess decay over suppression at the other end is simply due to the initial stages of recovery during the interval that separates conditioning and test, which curtails the measured suppression.

#### Increment detection

Systems that exhibit quantal release and deterministic inactivation feature increment detection, demonstrated for frog muscle (Fig. 7) but never explored with mammalian muscle. Successive incremental steps in voltage elicit successive peaks, same as increasing doses of IP_3_ in the studies that originated the concept (Muallem et al., 1989), manifesting the defining property of quantal systems as parcels with apparently different thresholds. This feature is found in the simulated flux record (Fig. 17), albeit with tamer kinetics than in the experiment (Fig. 7). In experiment and simulation, incremental detection was only found in an intermediate range of steps, separated by 5 mV in the experimental case, and in the simulation by (−)1.3 kT, or ∼9 mV.

### The simulated elementary events

#### Events at the couplon level

Our simulation derives events from a single couplon, in which channels are treated individually. Therefore, in addition to comparing ensemble averages with whole-cell flux, simulated events at the organellar (couplon) and molecular (channel) levels can also be compared with the experimental observations.

By contrast with the abundance of measurements of fluxes at cell level, couplon- and channel-level observations are both scarce (due to the difficulty in replicating the observations of coupled gating in bilayers) and sparse (i.e., difficult to perform under a variety of conditions). Thus, we expected only rough approximations in these comparisons.

In a recent review article (Rios, 2026), a necessary feature of any model of Ca^2+^ release in skeletal muscle was posited: that it be graded with voltage at the couplon level and therefore devoid of Ca^2+^ sparks (to match the experimental records of Fig. 3). The same article anticipated the difficulty to reconcile gradation with positive (allosteric) feedback. The present model complies with both requisites. The simulation equivalent of the individual couplon-level flux is the individual Markov run of the couplon. One such run, for a couplon activated by a 100 ms pulse of −4 kT (corresponding to depolarization to −54 mV) is illustrated in Fig. 18, representative of a larger set of realizations shown in Supplement 2. The blue trace graphs the flux through V channels (that is, simply the number of V channels open) as a function of time, while the red trace represents flux through the C channels, usually much greater, as it is equal to the number of open channels multiplied by 5 ($R_{C/V}$). The response to low voltage pulses in these cases is composed of brief bursts that seldom involve the whole couplon. In this qualitative sense, the simulated couplon-level events are not inconsistent with the elementary Ca^2+^ events shown in Fig. 3. The rough similarity extends to the events in frog muscle at very low voltages, which do not include Ca^2+^ sparks (and are therefore limited to the activation mechanisms of the mammal). See, for example, records at −72 mV in Fig. 2 of Shirokova and Ríos (1997).

For a quantitative analysis, we used Score (Nguyen et al., 2005), a measure of the “spark-like” quality of current from clusters, defined as the index of dispersion of the fraction of open channels (*N*_*O*_/2*N*)

$$Score\equiv \frac{Variance\left(N_{O}\right)}{Mean\left(N_{O}\right)}\frac{1}{2N}$$ (16)

which is >0.3 in spark records and spark-like simulations.

In the single couplon runs of Fig. 18 and Supplement 2, *Score* values range between 0.05 and 0.4, indicative of units that have high reactivity (visible in the tendency of channels to open in small groups), but fall short of producing sparks. A simulation of couplons without inactivation, in Discussion, will show that the high reactivity of the couplon—due of course to the allosteric coupling—is tamed by the strong inactivation processes.

#### The molecular-level comparison

**Figure 19.**
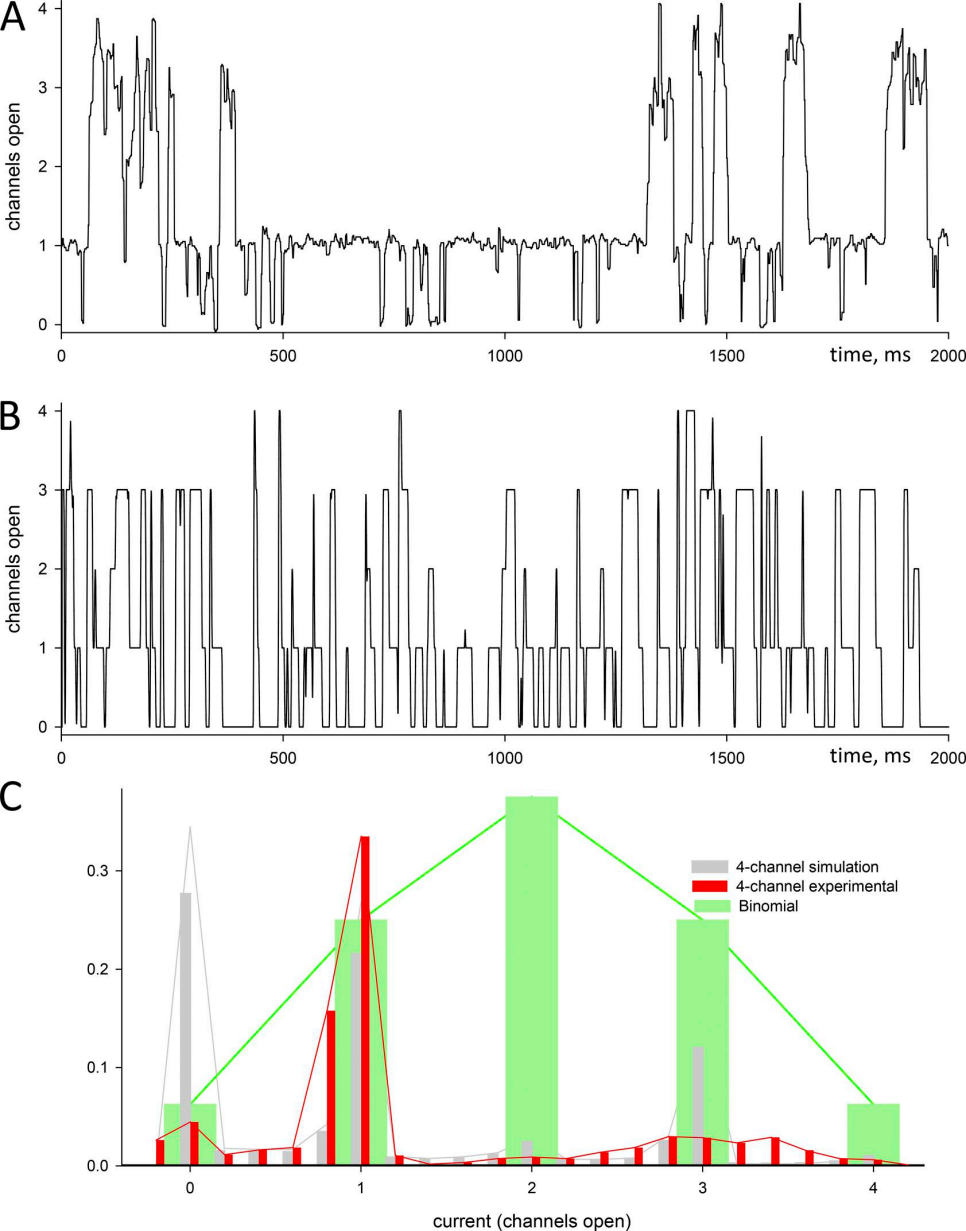
**Simulations with a 4-channel couplon compared with data in bilayers. (A)** Current from reconstituted rabbit RyR1 showing four levels by Porta et al. (2012), digitized from the original graph reprinted in Fig. 2 A. **(B)** Single realization of the Markov chain representing a 4-channel couplon. A low-pass filter (0.5 kHz) was applied, for better visual comparison with the experimental data. **(C)** All-points histograms of the experimental data (red), the simulation (grey), and the independent 4-channel set (“Binomial,” Eq. 17). Note the difference of both experimental and simulated distributions with the binomial, indicating robust inter-channel coupling. Also, that coupling in the sense used by Porta et al. (2012) of *simultaneous* or *quasi-simultaneous gating*, is intermittent in both experiment and simulation. And finally, that state *m* = 3 (three channels open) occurs more frequently than *m* = 2 in both experiment and simulation.

**Figure 20.**
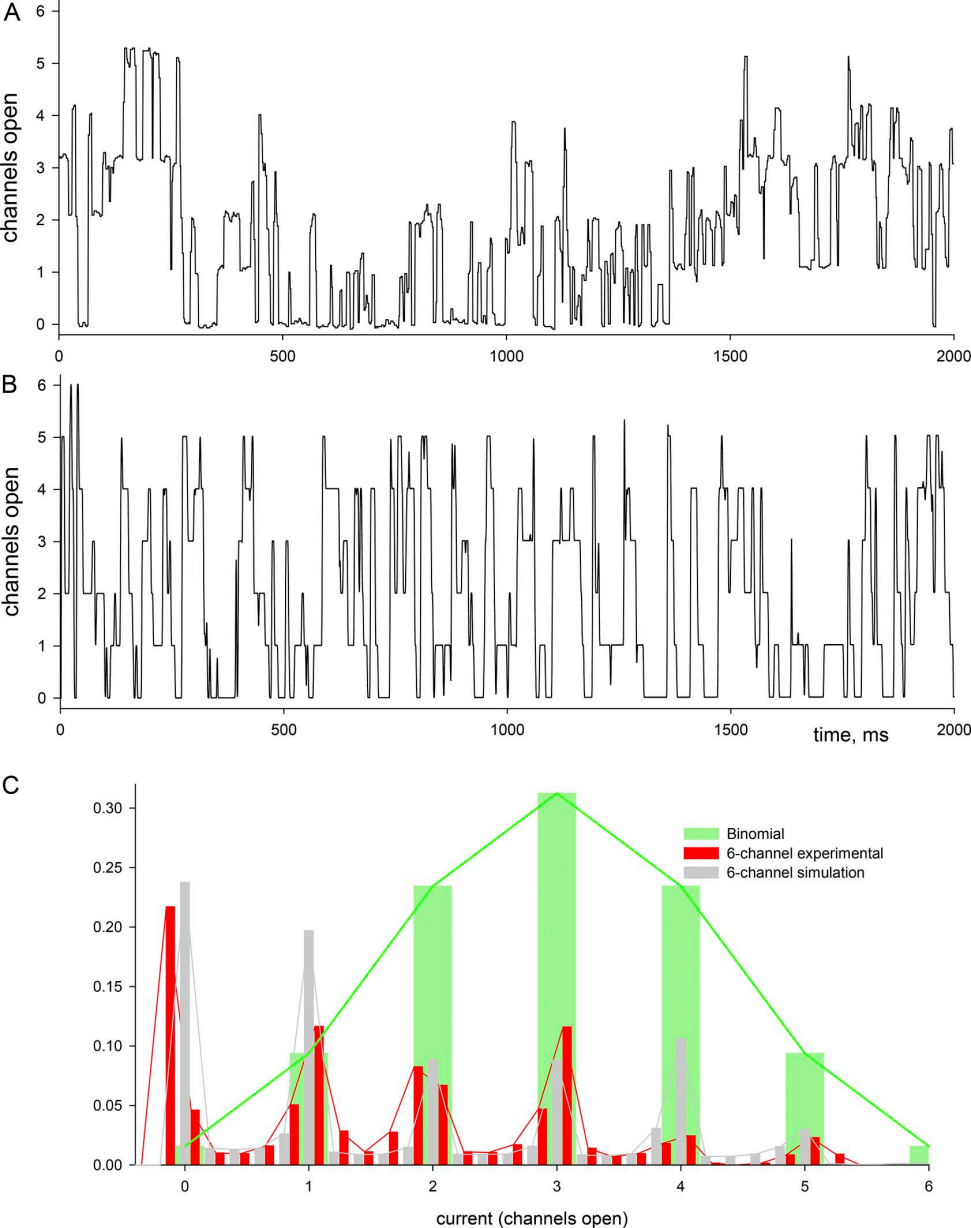
**Flux in a 6-channel model couplon and bilayer data. (A)** Current from RyR1 showing five or perhaps six levels (Porta et al., 2012), digitized from the trace in Fig. 2 B. **(B)** Single realization of the Markov chain representing a 6-channel couplon. A low-pass filter (0.5 kHz) was applied. **(C)** All-points histograms of the experimental data (red), the simulation (grey), and the independent 6-channel set (Binomial, Eq. 17). Again, the difference with the binomial indicates robust inter-channel coupling. The high frequency of state *m* = 3 remains in experiment and simulation, but is blunted in the latter (see text for explanation). Note that *m* = 6 did not appear the experiment and was exceedingly infrequent in the simulation, which is consistent with the interpretation of the experimental current as emanating from a 6-channel group.

Finally, simulations were made with couplons of four and six channels, intended to represent the coupled gating records of bilayer-reconstituted RyR1 channels. Among the few examples available, the records of Porta et al. (2012) have the greatest claim to physiological representativity for showing a dependence on ATP and Mg^2+^ typical of channels in physiological conditions. A simulated run of a couplon of four channels linked as #1–4 in Fig. 10 A is compared in Fig. 19 with a digitization (by a procedure detailed in Appendix B) of the experimental current stretch in Fig. 2 A (a current interpreted by Porta et al. (2012) as coursing through four channels). A corresponding comparison is illustrated in Fig. 20 between a simulated run of a 6-channel couplon and a digitization of the experimental current stretch in Fig. 2 B, presumably originating from six coupled channels (again, Porta et al., 2012).

The comparison required adjustments in the model. In well-polarized or long-term depolarized membranes, RyRs do not normally open. This is reflected in the model by intrinsic transition rates that combine for an (opening) equilibrium constant of 2,000^−1^. For channels to open when reconstituted in a bilayer, the inhibitions that keep them closed *in vivo* must have been somehow removed by the fractioning and reconstitution process. Ca_V_s, presumably removed by these processes, are the likely candidate inhibitor (Zhou et al., 2006). Their removal also eliminates any differences between V and C channels, which are entirely attributable to the Ca_V_ interaction. Thus, for the comparison, all four or six channels in the simulations are assumed to be equivalent; their current ratio is reset to 1, and the intrinsic gating rate constants are changed, so that the channels gate open and there is some flux to compare with experiments. This was achieved in the simulation by setting the intrinsic transition rates $k^{+}$ and $k^{-}$ to 0.125 ms^−1^ for a K of one and an individual channel $P_{O}$ of 0.5. All other model parameters were left unchanged. The digitized experimental records are in Figs. 19 A and 20 A. Representative runs of the model are in corresponding panel B. Panel C graphs the all-points histograms (in red and grey, respectively).

In both examples, observations and simulations have in common a wide departure from the distribution of states of $N_{T}$ (four or six) channels gating independently

$$P\left(m\;\text{open}\right)=\frac{N_{T}!}{m!\left(N_{T}-m\right)!}P_{O}^{m}{\left(1-P_{O}\right)}^{N_{T}-m}$$ (17)

represented in both figures by the green bars. The divergence confirms that the channels in the experiments are coupled. Qualitatively, as already observed by Porta et al. (2012), the “coupling”—in the restricted sense of channels actually opening or closing together—is variable and intermittent. The simulations share this feature. By contrast, they are conspicuously inconsistent with the type of obligatory coupling seen in the records of Marx et al. (1998) (The discrepancy among the various degrees of channel coupling in published work was reviewed by Rios [2026]).

Most suggestive, however, is the prevalence of states m = 1 and *m* = 3 (i.e., one or three channels open) in both stretches of experimental current, a feature again shared with the simulations. The prevalence of m = 3 is surprising and suggestive; indeed, there are not many ways in which a set of four channels can be linked so that the probability of three channels open is greater than that of 2. In the model 4-channel couplon (which can be envisioned from the illustration in Fig. 10 B), one channel is linked allosterically to two others, which predicts that when two are open, the one linked to both will experience a substantial nudge to open as well. As shown with Fig. 20, the preference for the open trio is less evident, in model and experiment, with 6-channel clusters, presumably diluted there as two channels, #3 and #4 (Fig. 10), acquire three linked “partners,” which promotes states with m = 4.

The prevalence of m = 3 in small coupled sets could therefore be understood as a marker of skeletal-couplon-style allostery. (The conditional “could” here is necessary, as the inference is based on the single observation by Porta et al., 2012).

## Discussion

A novel model of Ca^2+^ release flux in mammalian skeletal muscle is presented with a core idea: C channels are activated via allosteric V–C coupling.

Buttressing the assumption is the regularity of the skeletal muscle couplon structure, remarkably unlike the variable clustering in cardiac muscle (Rios, 2026) and set so firmly that it resists various fractionation and extraction procedures. What valuable physiology could emerge from a highly conserved *regular* assembly of channels? Features like positive feedback, synchronicity and production of high local concentrations of released Ca^2+^ can be achieved by simple clustering. Only allosteric coordination also requires regularity. Putting this inference together with their lack of other means of activation, we conclude that C channels are either activated allosterically or not at all.

Here, we review the defining aspects of the model and how its output compares with experiments and describe novel insights gleaned from this work.

### A simple model, built from structural and functional evidence

The model stands on five assumptions; four are strictly derived from observations: (1) Its basic “anatomy”—the interlaced, stoichiometric linkage of channels—is derived from the couplon’s checkerboard structure. (2) That channels V are activated by Ca_V_s while C are not stems from evidence that only V channels contact Ca_V_s (Chen and Kudryashev, 2020; Xu et al., 2024). (3) That CICR does not contribute to activation is now a consensus (e.g., Ríos, 2018; Kobayashi et al., 2025). (4) That inactivation depends solely on channel state (hence inure to the varying [Ca^2+^]_cytosol_) is based on evidence reviewed with Fig. 8. Only a fifth feature, that these two sets of channels contribute separately the two kinetic components of the flux waveform, has no experimental support. It is assumed for its simplicity and affirmed by the model’s predictive success, by contrast with the failure of the alternatives (cf. Appendix C).

The model is simple: a statistical ensemble of identical couplons, each with identical channels differing only in their external controller contact.

The sparsity of components combines with a limited repertoire of channel states (two for V channels, four for C channels) to result in a model with 12 parameters, which does not add up to a full 12-dimension parameter space as the values of some variables are highly correlated or bound narrowly by observations. (Thus, the intrinsic transition rates *k*^*+*^ and $k^{-}$ must differ by a ratio of thousands to keep channels closed at rest; the first inactivation rate must be near 3 ms to match the fast decay from peak flux; the recovery rates are highly correlated with the inactivation rates, the kinetic distribution factors *ν* and $v_{V}$ are unlikely to lay outside the 0–1 range, and the ratio of channel currents *R*_C/V_ is constrained by the observed Peak/Steady ratio). The variables that adopted unexpectedly high values are the allosteric energies $\varepsilon_{OO}$ and $\varepsilon_{CC}$, but these are high only by comparison with values of convenience used in the proof-of-principle studies of Stern et al. (1999) and Groff and Smith (2008). Adding to the simplicity, the number of channels per couplon may vary without changing the output significantly (Supplement 1).

That a small channel cluster with a simple set of rules matches many properties of the macroscopic flux and its elementary events suggests that the model meaningfully represents physiological processes.

### The model adheres to physical principles

Channel gating interactions (electrical and allosteric) are described as energy transfers biasing the open-close reaction in a statistical ensemble, with state distribution determined by Gibbs free energy. These energy transfers derive from a known source, the electric field, in amounts consistent with the electrical work that mobile charged residues can extract. The allosteric transfers are reciprocal, satisfying microscopic reversibility, while electrical transfers are not, consistent with their irreversible effects. Finally, the decisions at the individual channel level are stochastic, with probabilities determined solely by interaction energies and mass action.

### The experimental observations are matched

The model simulations reproduce multiple aspects of the flux of Ca^2+^ release in rodent fast twitch muscle fibers. The match includes the voltage dependence of Ca^2+^ release, its mid-voltage, and effective valence. The scale of the simulated flux is consistent with experience, inasmuch as it involves every channel in the couplon, which either opens or inactivates at the higher voltages. It emulates the transient and steady phases of flux kinetics, the ratio between peak and steady values, and its shallow dependence on pulse voltage. The kinetic parameters are reproduced within the experimental variance. The simulations also matched with quantitative accuracy the time course of recovery after inactivation and features of “quantal release” and “deterministic inactivation” further discussed below.

The simulated Ca^2+^ release satisfies a characteristic of flux in mammals: individual couplons respond incrementally to increasing *V*, unlike cardiac couplons, which respond in all-or-none manner, with Ca^2+^ sparks.

Both in the 4- and 6-channel cases, the simulations matched the variable degree of channel synchronization found in bilayer recordings. The most telling agreement was found in the higher frequency of the three-open channel state, which emerges as a hallmark of small groups of RyRs, linked skeletal-muscle-style.

### The fit adds mechanistic information on gating

The model flux activates with voltage with a Boltzmann slope factor of 8.5 mV, which is consistent with the reference value (Ferreira-Gregorio et al., 2017). More interestingly, the limiting logarithmic slope at low voltages, 3.91 mV, was consistent with the value by Pape et al. (1995), 3.7 mV—a sensor with effective charge of ∼6.7 *e*. From this value and evidence that only one of the four domains of Ca_V_1.1 contributes to release activation and only two residues cross the membrane field (Pantazis et al., 2014; Pelizzari et al., 2024; Heiss et al., 2025; Angelini et al., 2025), the effective valence would be 8 *e* if the threshold stoichiometry (minimal number of sensors that must cross the membrane field to activate the connected RyR tetramer) were four. Current work of Brum and Rios, unpublished, is consistent with a more flexible stoichiometry of three, which would bring the effective valence to the observed 6 *e* -to-7 *e* range.

### Quantal release and deterministic inactivation are explained

While quantal release and its consequent deterministic inactivation were first defined as emergent from a set of channels/receptors with different sensitivities, the present modeling recreates those properties with a *homogeneous* set of channels. Classical inactivation, which fails to match quantal events, occurs regardless of activation; for example, if inactivation is induced by bulk cytosolic Ca^2+^ and therefore can affect closed as well as open channels (Schneider and Simon, 1988; Simon et al., 1991). The critical feature that leads to quantal properties in the model is that inactivation is linked to activation; it only affects open channels.

### A proposed role of Ca^2+^ is endorsed

A Ca^2+^-mediated mechanism for inactivation obligatorily linked to channel opening arises if inactivation requires binding of the ion to a site with affinity sufficiently low that it can only be satisfied by the [Ca^2+^] reached near the open channel mouth. W.K. Chandler’s laboratory in fact provided evidence for an inhibitory Ca^2+^-binding site, located at a distance of 20 nm or less from the channel mouth—a distance that puts the site within the same RyR tetramer (Pape et al., 1998). The low affinity of the hypothetical site is also consistent with the high [Ca^2+^] required to inhibit channel opening in bilayer reconstitution experiments (Ma et al., 1988; Sánchez et al., 2003; Laver, 2018).

### The absence of CICR gains a mechanistic basis

RyRs of the skeletal isoform 1 are intrinsically sensitive to activation by cytosolic Ca^2+^ (Murayama and Kurebayashi, 2011; Ríos, 2018). As such, they produce large collective events *in situ*, albeit sufficiently different from Ca^2+^ sparks that they were dubbed ECRE (Kirsch et al., 2001), and do so only when the plasma membrane is removed. In fact, the muscle fibers that produced the elementary release events illustrated in Fig. 3 did exhibit ECRE, but only in the side pools of the Vaseline-gap assemblage, where the fiber membrane is destroyed (Csernoch et al., 2004). For these and other reasons (e.g., Zhou et al., 2006), the absence of activation by Ca^2+^ under physiological conditions is attributed to control by the coupled Ca_V_s. The inter-RyR connections in the present model provide a way whereby Ca_V_s suppress CICR not just in the underlying V channels but, by allostery, also in the C channels.

### A highly reactive couplon

**Figure 21.**
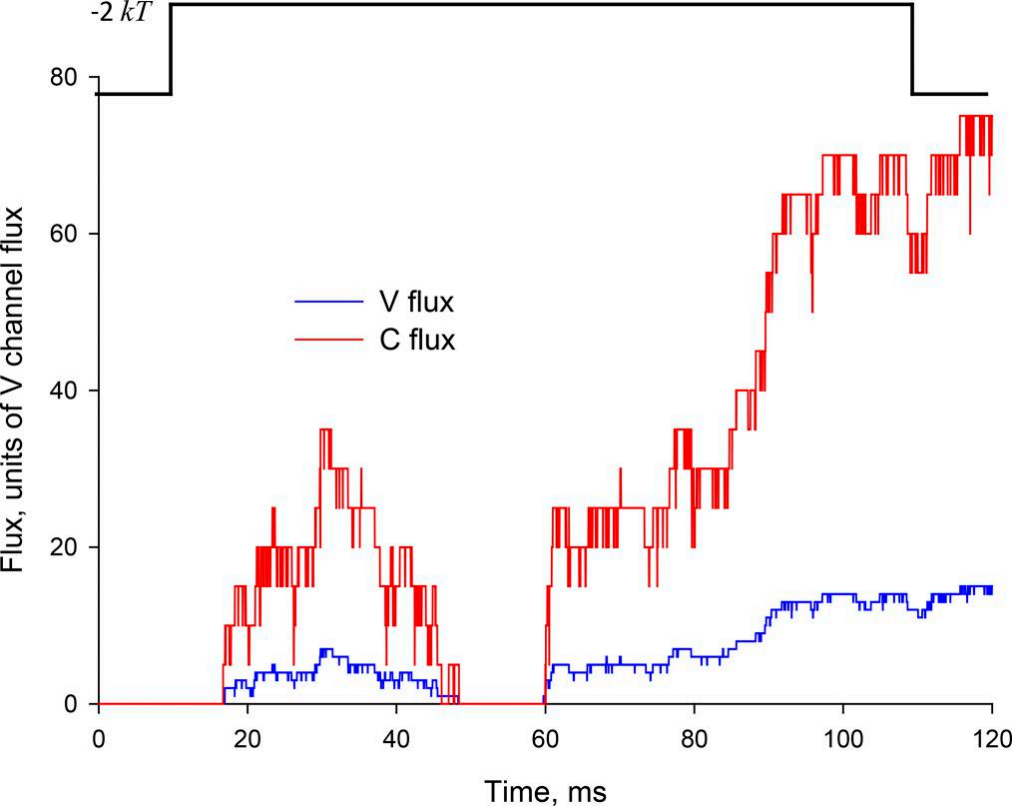
**The couplon in the absence of inactivation.** Single Markov realization with a setting similar to the simulation in Fig. 18, except for the absence of inactivation (elimination of states $I_{1}$ and $I_{2}$–see Fig. 10 A). Note that by the end of the pulse almost all channels in the couplon were open. A similar tendency was observed in replications of the Markov run, with multiple examples in Supplement 2 (Fig. S2 2).

When examining the simulated couplon-level elementary events—single Markov runs in Fig. 18 and Supplement 2—we found that their *Score* values did not usually reach spark levels but were close, consistent with a highly excitable couplon. Here, we show that the high reactivity is due to the allosteric interactions. To identify properties due to allostery alone, simulations were done with a version of the model that lacks inactivation (referring to Fig. 10, the state diagram is made the same for both V and C channels, consisting only of states C and O). Fig. 21 graphs the flux elicited by a very low energy pulse (− 2 kT or 14.3 mV from the holding potential) in a representative Markov run. Even at this low voltage, the activation eventually invades most of the couplon. This is true of many individual runs, a larger sample of which is in Supplement 2. The typical *Score* values oscillate between 0.02—for runs that fail to activate most channels, and 0.55—for runs that overtake the whole couplon. Thus, the partial model, with activation by voltage and allostery but without inactivation, results in an overly excitable and poorly controlled couplon. This is a reflection of the values of $\varepsilon_{OO}$ and $\varepsilon_{CC}$, very high compared with the precedents in Stern et al. (1999) and Groff and Smith (2008). Stability and control graded with voltage only emerge in the presence of robust (fast and fatal) inactivation.

### A plausible answer for a question of voltage sensing

The good match between the dependence *F (V)* in model and experiments came as a surprise because it was not sought specifically. In optimization runs, we simply kept increasing the free energy $\varepsilon_{V}$ added during the activating pulse, until the couplon activation saturated, at −14 kT. No other fitting was done. A holding potential of −90 mV and a saturation potential of +10 mV (Ferreira Gregorio et al., 2017) establish the correspondence with experiment at −7.14 mV/kT. With this calibration, the model *F* acquires a Boltzmann dependence on V (Eq. 1) with similar parameter values as those of the experimental reference, with the implication that the effective valence of the sensor is also met by the model.

This agreement has a corollary. The energy required for activation comes from the electric field, as work done on the mobile charge of the sensors. As argued earlier, the upper bound of the electric work per RyR can be set with some confidence at 30 kT*.* The RyR gating task as modeled (a reduction of Δ*G* by at most 14 kT) is therefore doable with the energy resources at hand. But mustering the full 30 kT, which would leave some margin for other costs of Ca_V_’s activation, requires four Ca_V_s per RyR. Though a bit tenuous, this is a first justification for the presence of four Ca_V_s per connected RyR1 in the T-SR junction.

### Concerns

#### Mismatches

There are mismatches between model simulations and observations. A salient one involves the excessively fast rise in permeability/flux that the model produces at high applied voltages, when channel opening becomes essentially instantaneous. By contrast, recorded times to peak have limiting values of ∼3 ms in experiments tuned for clamp speed (Ursu et al., 2005). Part of the discrepancy is due to the simulations’ instantaneous change in voltage, contrasting with its finite rate of rise in experiments. Indeed, at our request, Prof. W. Melzer estimated the time to half voltage change in the T membrane by the time to peak of the capacitive charging transient, at 1 ms in the best cases recorded by Ursu et al (2005). Alternative simulations of flux, in which the voltage pulses have an exponential rise with 1 ms time constant, documented in Appendix C, have times to peak lengthened by between 1.5 and 2.5 ms, depending on voltage, thus bringing the simulated and observed values closer. Another source of experimental lag is the time required for the movement of the VSDIII, which determines gating in RyRs (Pelizzari et al., 2024; Angelini et al., 2025), 2–3 ms in electrofluorometry experiments of Angelini et al. (2025). In sum, the known causes of experimental lags are sufficient to reconcile the fast onset of Ca^2+^ flux in the model with that in the observations.

#### Systematic excess suppression

A systematic excess suppression, by as much as 20%, is also found in the simulations over the simple rule of deterministic inactivation (suppression equals decay) when the conditioning voltage is much lower than that of the test. Additionally, at high conditioning voltages, suppression may be 20–30% lower than decay (Fig. 16). Again, a strict equality was never observed in experiments; the deviations, small but systematic (Fig. 6), were in the same direction and ranges as those in the simulation (Fig. 16).

#### Inactivation is also deterministic in other muscles

Quantal release and deterministic inactivation are present in the frog. How can frog couplons, with their added endowment of CICR-activated RyR3, share these properties with the simpler mouse couplons? Deterministic inactivation emerges from its obligatory link to activation. That both types of couplons have it suggests that the obligatory link to channel opening is shared by both RyR isoforms of the frog couplon, even if activation is allosteric for RyR1 and Ca^2+^-mediated for RyR3.

#### Extreme simplification of the effects of voltage

It is easy to envision a control by the DHPRs more complex and subtle than that described in the model. For example, the action of the four DHPRs could be cooperative, mutually reinforcing, which would result in a different voltage dependence of activation. Similarly, the multiple interactions of V channels could conceivably bias their intrinsic gating parameters differently. All these possibilities may be explored quantitatively if further tests demonstrate systematic prediction errors; at this point, they only add value to the simple assumptions that make the model work.

#### A teleologic speculation

The optimized value of one model parameter, $R_{C/V}$, detracts from the model’s credibility. Two channels, C and V, identical but for the interaction with Ca_V_s and associated proteins, are proposed to differ in conductance by a large factor. How, by what mechanism, can this difference be imposed, and why was it favored by natural selection? The answer may lie with the advantage for mammals of having a large Ca^2+^ flux that is always under fast voltage control.

The speculation requires a preamble: in skeletal muscle of zebrafish and amphibians, CICR is produced by parajunctional arrays of RyR3 (Perni et al., 2015). Long ago, Elizabeth Stephenson volunteered (verbally, in front of the poster that prefigured Shirokova et al., 1996) a reason for *not* having CICR as follows: its loss, and absence of RyR3 in mammalian skeletal muscle, is paired to having two triads per sarcomere, located at the sarcomeric regions where Ca^2+^ binds to troponin, by contrast with just one in cardiac and frog skeletal muscle, farther out by the Z disks. This feature, said Stephenson, allows mammalian muscle to generate the needed [Ca^2+^] gradients with lower, better controlled flux—and fewer isoforms.

Stephenson’s argument explains the adoption of the less hazardous non-CICR mechanisms in mammals. But still, why the V-C duality instead of controlling every channel by voltage? As stated before, there is firm evidence that the suppression of CICR in RyR1 appears together with RyR control by voltage in embryonic development and is therefore due to their interaction with Ca_V_s (Zhou et al., 2006). Perhaps the interaction also limits RyR conductance, by simple crowding, and/or a conformational umbrage imposed allosterically by the physical contacts with multiple voltage controllers. 8.8525mm In this speculative view, Ca_V_-free channels would be useful for having high conductance configurations unavailable to the Ca_V_-interacting ones. That reconstituted RyR channels exhibit substates, including some with ∼1/4 the full conductance (Fill and Copello, 2002), gives some basis for the idea, admittedly strange, that half of the RyRs are physiologically limited to states of lower conductance.

### Applications

The accuracy of this model’s predictions makes practical a computational module that can be incorporated to bigger, more comprehensive models of physiological processes. A sequential development of extensions that use this module, already started in our labs, may progress through the following stages: (1) Ca^2+^ flux elicited by recorded action potentials and their trains; (2) flux exiting from a realistic SR, which would include depletion and other intra-SR changes resulting from it—for example, calsequestrin depolymerization and STIM activation; (3) whole-cell models, which could incorporate multiple couplon sizes, cytosolic Ca^2+^ buffering, plasma membrane electrophysiology, and Ca^2+^ transport; (4) explicit spatial distribution, with radial conduction and the steep Ca^2+^ gradients present *in vivo*; (5) contractility, initially isometric, and accounting for metabolic demands; (6) pharmacology in conditions of compromised RyR or electrophysiological function. Experimental observations accumulated over decades provide comparison terms for every step. The validated models could then be applicable to conditions that cannot be realized in experimental settings.

### A synthesis

This model pictures macroscopic Ca^2+^ release in mammalian muscle as a function optimized for speed and tight control. Unlike cardiac or amphibian skeletal muscle, this process is locally “smooth” (lacking sparks), hence more efficient. Ca^2+^ release is the job of a homogeneous set of couplons. These consist of a regular double row of tetrameric RyR channels, identical but for the connection of the V class to Ca_V_s, in interlaced quartets where one V links stoichiometrically to three C, and vice versa. The result is a braid-like device, mechanically resilient, physiologically intricate, and mathematically nonlinear. Two couplon-containing triads per sarcomere are positioned at regions of myofilament overlap. This arrangement removes the need for the potentially explosive CICR mechanism. Activation is led by V channels operated by linked Ca_V_s and followed by C channels via reciprocal allosteric connections. Because these connections are spatially interlaced, the couplon becomes an allosteric continuum, ensuring high reactivity. Fast and fatal inactivation keeps this excitable device under strict voltage control. Control by apposed Ca_V_s is dual: it activates the device while simultaneously blocking CICR—a block extended to C channels through allostery. Because the interaction with Ca_V_s reduces the current-carrying capacity of V channels, the C class becomes necessary to provide full channel opening. Finally, the presence of four Ca_V_s per RyR tetramer provides sufficient mobile charge to extract the electrical energy required to reform the underlying V leaders and its C followers.

Many statements in this synthesis can be doubted, but none has been disproved so far. Together, they paint a consistent picture of an elaborate and essential device.

## Appendix A: A determination of errors in the simulation of Ca^2+^ flux

The “error” in our averages of stochastic runs of Markov chains is defined as the probable difference between the average value presented and its “true” value, which cannot be other than the average in the limit of an infinite ensemble of runs. An analytical study of error is made difficult here for the convergence of six transitions, all treated stochastically, operating asynchronously in an initial group of 60 channels. A semiempirical determination is presented instead. The inherent error of the averages (functions of voltage/energy and time) is first analyzed for individual runs, at every time point in the simulation. From this time point–dependent, single-run error, a collective error of the average at the given point is derived. In turn, the time point–dependent error of the average is used to calculate errors in emergent measures.

**Figure AA1.**
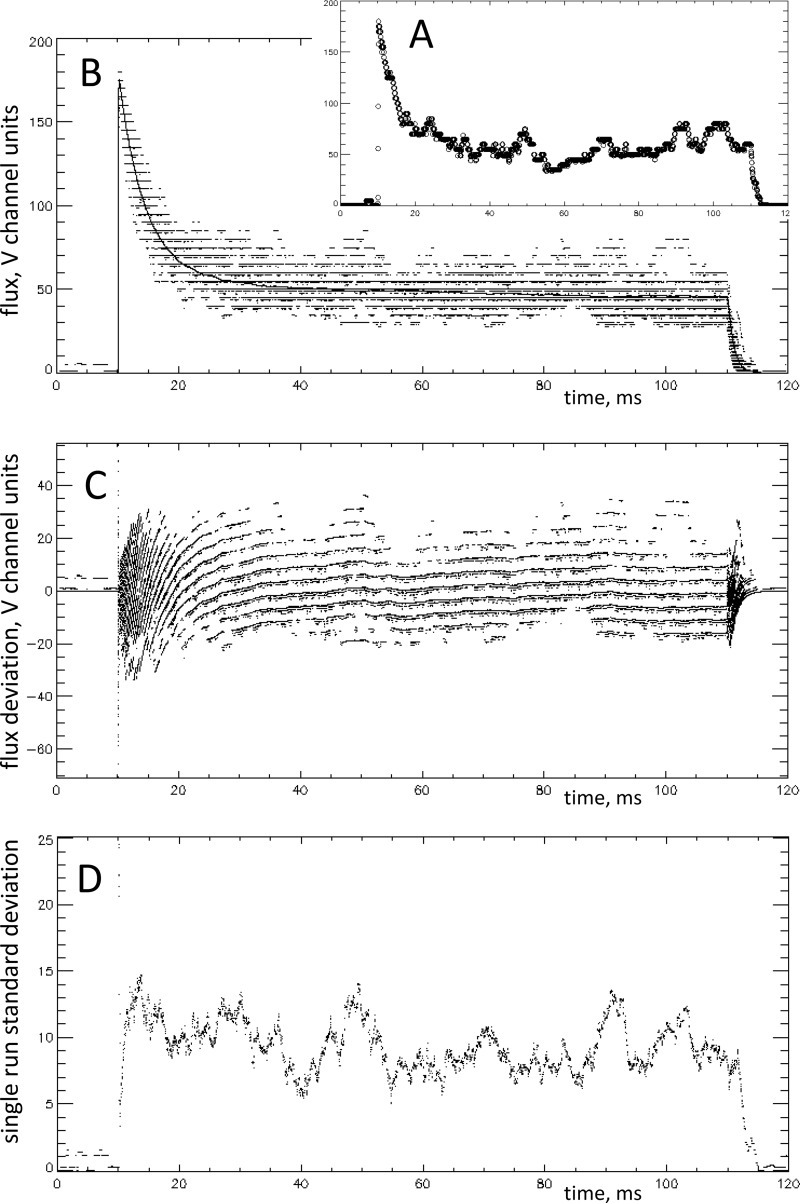
**A semiempirical calculation of simulation error.** See description in text.

An initial evaluation of error of the individual runs assumes that the error of the averages (of 800 or 6,400 runs) is negligible by comparison and uses the averages as true reference. An individual run of the response to a large depolarizing pulse is plotted in Fig. AA1 panel A. 20 such realizations are plotted in panel B, together with the 800-run average. (Note that the individual runs only adopt integer values). Because individual runs are independent, the standard deviation of the individual run is estimated at every time point, as the standard deviation of the sample of 20 runs shown from the 800-run average. The individual differences are in panel C, and their standard deviation is plotted in D for every time point.

As estimates from a limited set of runs, these standard deviations have their own large error, which could be reduced by enlarging the sample. But it is clear that during the voltage pulse the standard deviation does not have a trend. Therefore, we take their average value during the pulse, 9.78 V channel flux units, as a good estimate of the approximately time-independent standard deviation of the individual runs. Clearly this measure has errors; possible changes during the pulse, errors in the average—which is not a true reference—and others. But none of those seem large enough to compromise the calculation that follows. Namely, if the standard or probable deviation in the individual runs is about 10 units, then the standard error of their mean should be about 10 divided by the square root of 800 or 0.35 units. This is an estimate for the individual time point. Obviously, the error of collective measures, like steady flux, decay or recovery time constant, will be less than that, and not relevant to the main conclusions of the study.

As for the simulations of flux elicited by very low voltages (Fig. 14), a rough upper bound of error can be calculated as the ratio of the same estimate of standard deviation in individual runs, 10 units, and the square root of 6,400. The result, 0.15 flux units, is an upper bound of the error because the individual run error decreases at lower voltages. In any case, again a probable error of 0.15 units or less does not alter the agreement between simulation and experiment at low pulse voltages (Figs. 5 and 14 in main article).

## Appendix B: A procedure to digitize records from analog graphic plots

**Figure AB1.**
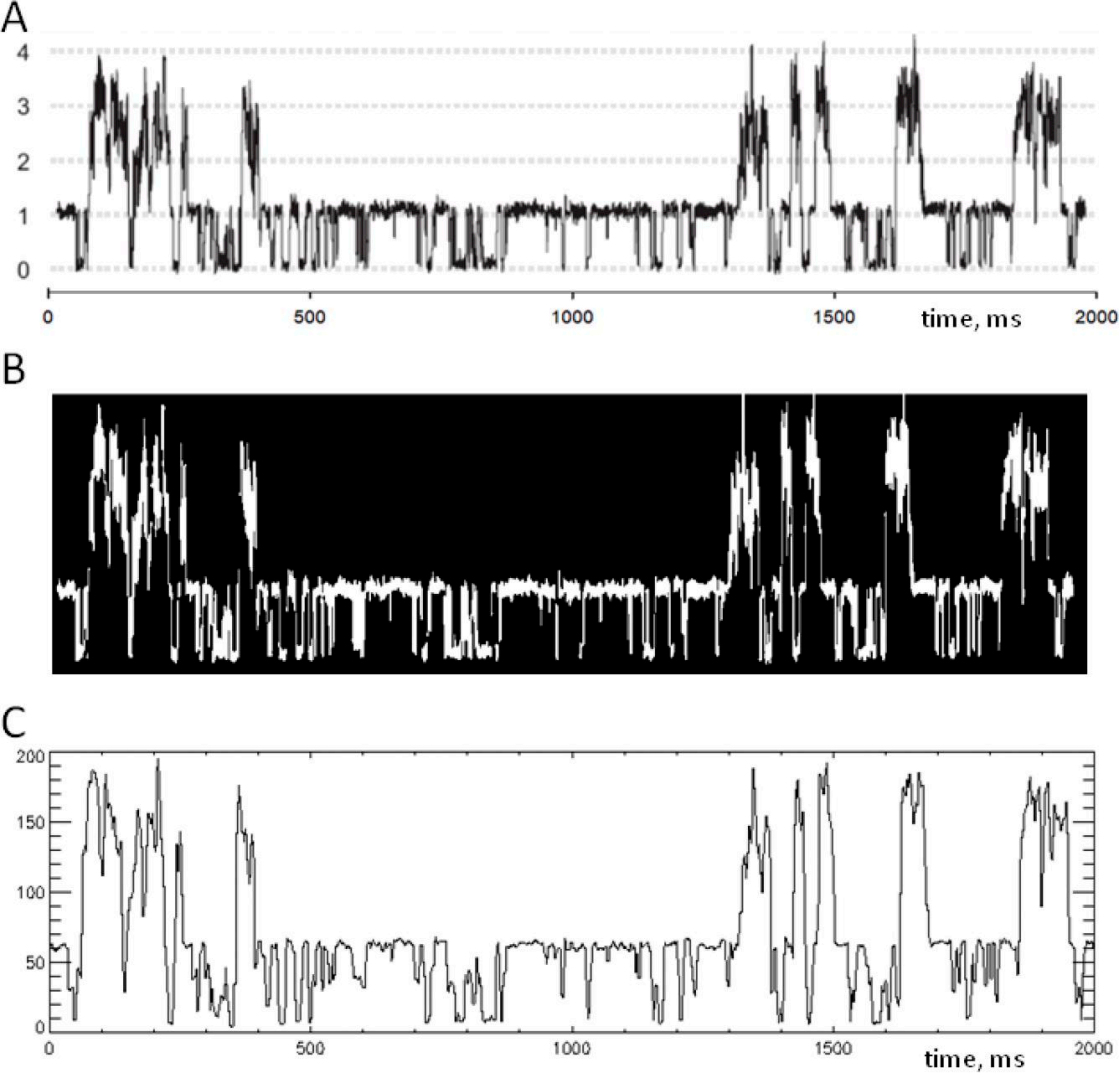
**Example of the digitization process. (A)** Source image, in PNG format. **(B)** TIFF formatted by ImageJ. **(C)** The digitized variable “*yt,*” output of program below.

The simple procedure starts from a graphic image of a plot, in PNG or another common format, as the example in Fig. AB1 panel A (from Porta et al. [2012]). The image is opened in ImageJ where it is processed as follows:

Open PNG. Dropdown menu *Process*. Choose *Binary*. *Mask*. Rectangle mark region of interest. *Crop*. Output as tiff. (“banks.tif” in the programming example). The mask image is graphed in Fig. AB1 panel B.

The process is completed by the simple IDL program in the text box. The resulting function *yt* (*time*) is output as a text file in two columns. It is plotted in Fig. AB1 panel C.

**Box AB1.**
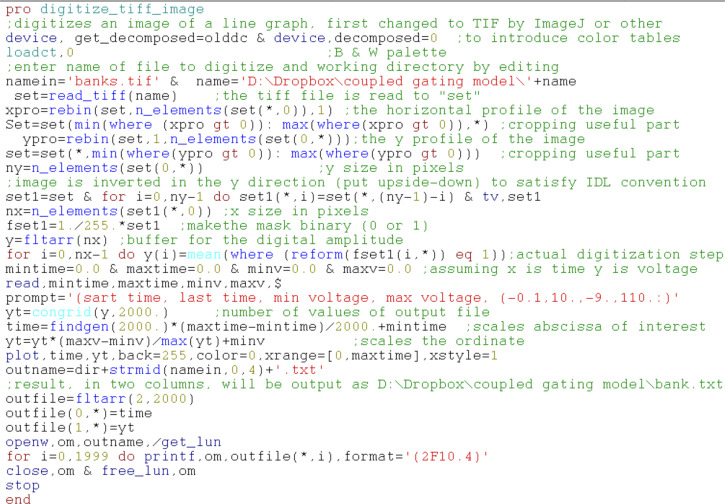
Simple digitization program.

## Appendix C: Alternative versions of the model

### AC.1: Model with a single inactivated state

**Figure AC1.**
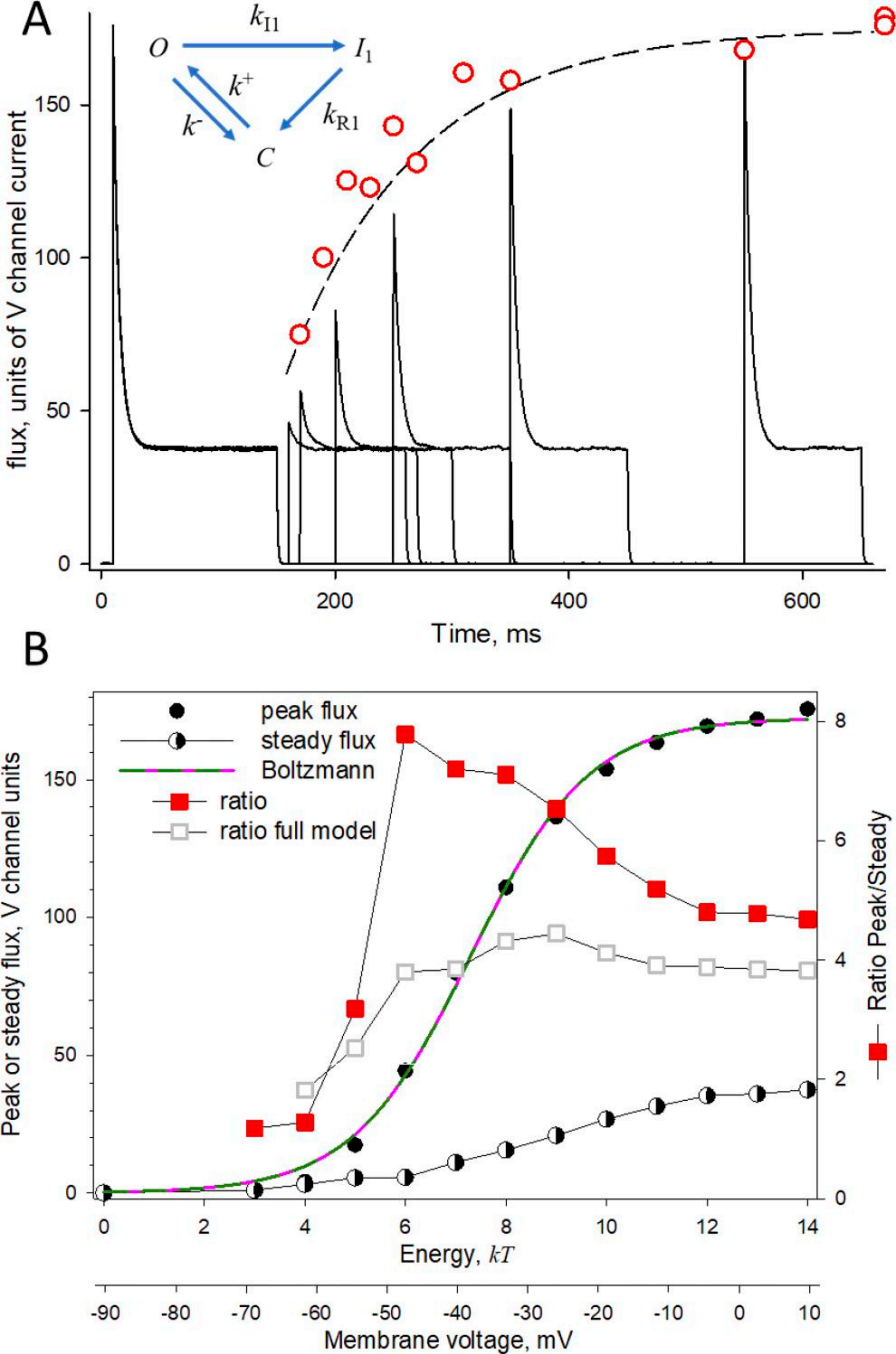
**Properties of a couplon with a single inactivated state accessible to C channels.** **(A and B)** Flux (A) and kinetic parameters (B) in simulations of a model with a single inactivated state represented in diagram at top. Model parameters values are those listed in Table 1, except for rec1_ht, changed to 80 ms in the present case. **(A)** The red circles plot measured peaks of the test pulse response reported by Sárközi et al. (2000). The dashed curve is the best exponential fit to the recovery of the peaks in simulations with the preferred model, i.e., the same curve plotted in Fig. 15 of the main article. **(B)** Peak ($F_{P}$) and steady ($F_{S}$) values (circles) and their ratios (red squares). Open squares plot ratio $F_{P}/F_{S}$ in simulations with the preferred model, reproduced from Fig. 12 in main article.

The simulations matching an experiment of recovery from inactivation, illustrated in Fig. 15 of the main text, were copied with the model modified according to the diagram in Fig. AC1 A, that is, eliminating state I2.

Panel A in Fig. AC1 plots the simulated fluxes elicited by pairs of pulses at different intervals. To match the data of Szentesi et al. (red) almost as well as the original simulation (dashed curve), the rate of recovery from inactivation had to be slowed (*rec1_ht* = 80 ms, compare with 20 ms in the original, Table 1), while all other parameters remained the same. Panel B plots peak and steady flux levels and their ratios, as a function of voltage. The ratios, in red, fail to match the rise to a sustained level observed in most experiments and reproduced well by the original model (open squares). Interestingly, the Boltzmann fit to the peaks of the modified model (multicolor curve) was nearly identical to the fit of the original simulation (which in turn was a good fit to the experimentally observed voltage dependence). Further exploration of the parameter space failed to match the trio of observations (i.e., time course of recovery, voltage dependence of ratio, and voltage dependence of peaks).

### AC.2: Models that assume equal currents through open C and V channels

**Figure AC2.**
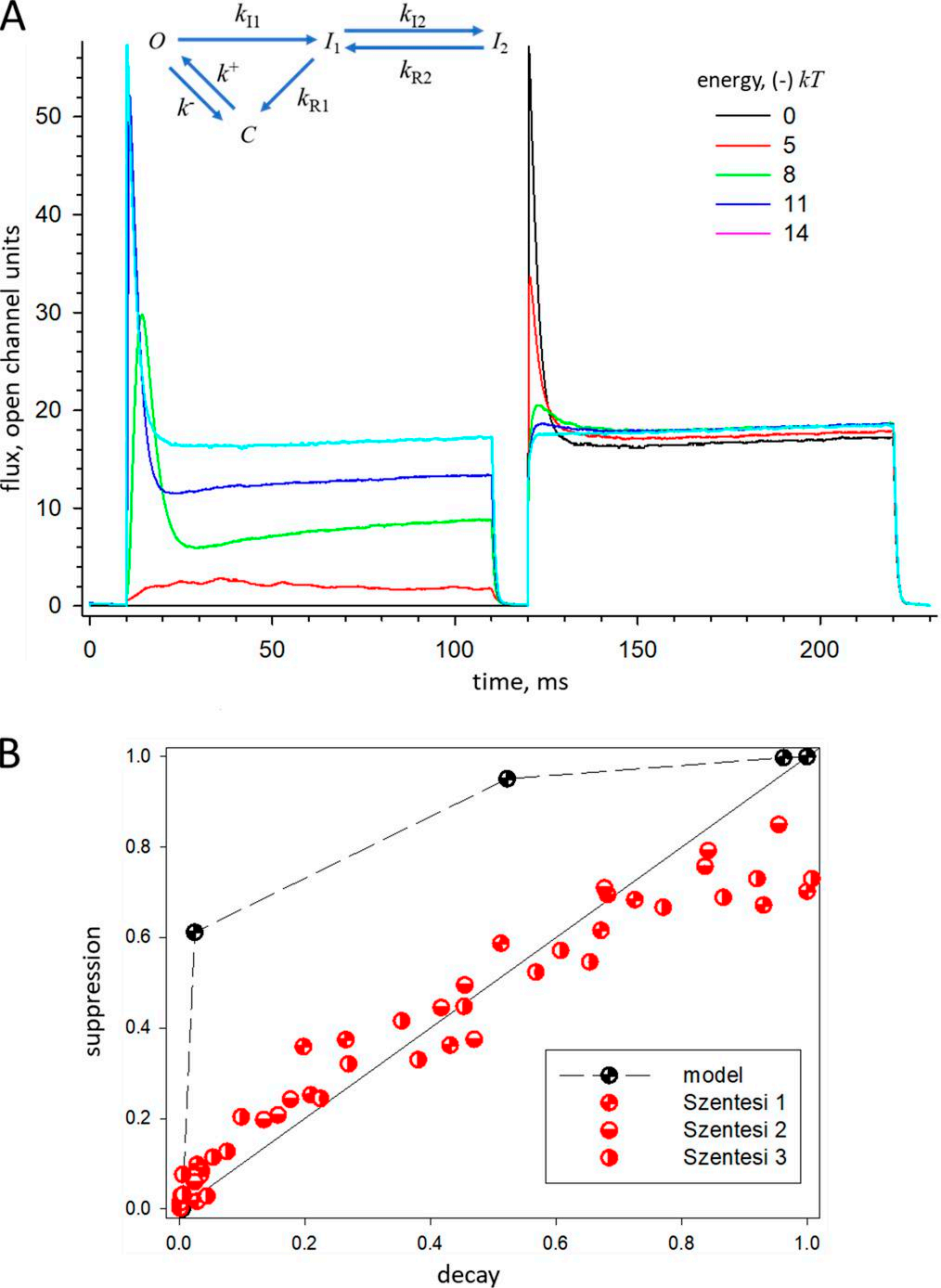
**Properties of a couplon with equal C and V open channel currents.** Both classes of channels follow the state diagram at top, identical to that of C channels of the preferred model in the main article. Parameters are those listed in Table 1, except rec1_ht, increased to 100 ms (a change needed to achieve flux with a large inactivating component). **(A)** Simulations of flux. elicited by paired pulses (conditioning and test), implementing variable conditioning on a large test, as in Figs. 6 and 17 of the main article. **(B)** Decay and suppression measured on the simulations in A. The red symbols represent experimental observations.

**Figure AC3.**
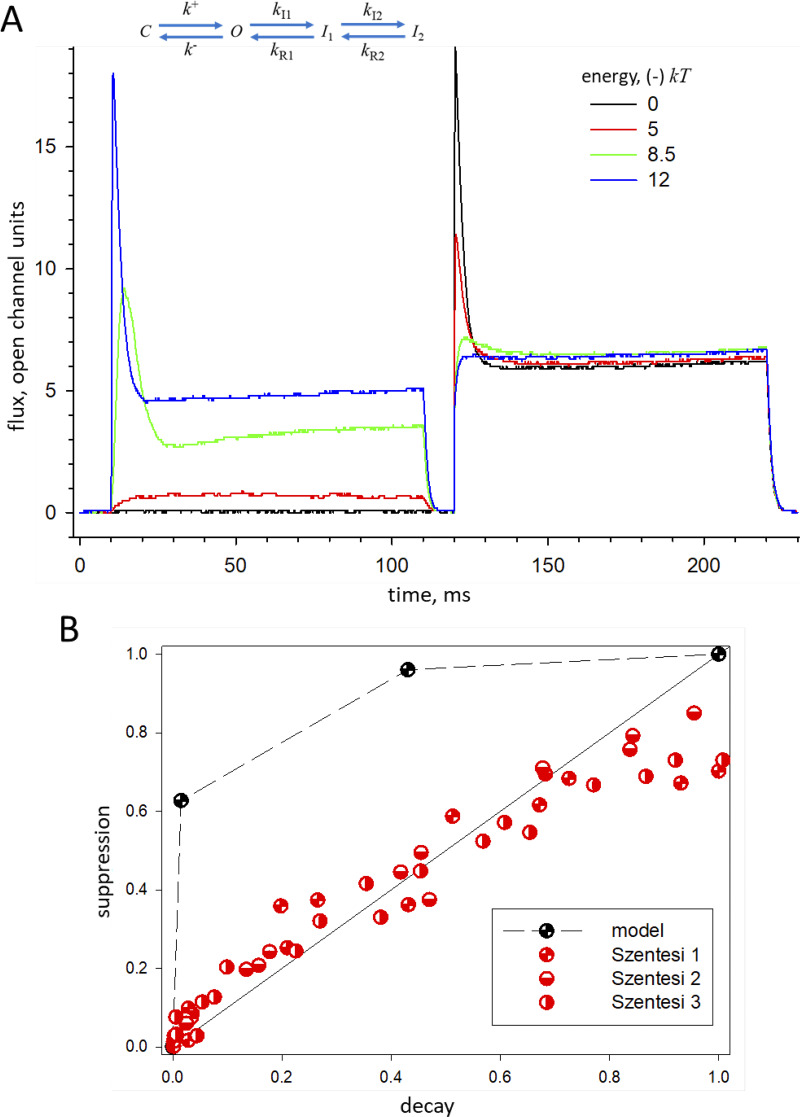
**Simulations of flux elicited by paired pulses in an alternative couplon with equal open channel currents.** **(A)** Both V and C channels follow the state diagram at top (A), which differs from that of the preferred model in the main article in that inactivated channels recover to the open state. Parameters are those listed in Table 1 of the main article, except rec1_ht, increased to 100 ms (to achieve flux with a large inactivating component). **(B)** Decay and suppression in simulations of the effect of variable conditioning on a large test, as in Figs. 6 and 17 of the main article. The red symbols represent experimental observations.

A first subset of simulations assumed that the decay from peak to steady level is due to partial inactivation affecting both C and V channels. With this common assumption, two alternatives were tested: either the inactivated channels recovered to Closed (C), as in the original model, or to Open (O). Simulations with both are illustrated with Figs. AC2 and AC3, respectively. To add generality to the examples, Fig. AC2 illustrates a simulation with a 60-channel couplon (30 C and 30 V) and Fig. AC3 a 20-channel couplon.

While these alternative models were tested with the full set of pulse configurations explored in the original, the most marked mismatch was their failure to exhibit the quantal properties illustrated with Figs. 6, 7, and 17 of the main text. Fig. AC3 illustrates simulations with the assumption of equal open channel flux for C and V, with both classes recovering from inactivation back to the Open (O) state, as in the diagram at top.

### AC.3: Why do the alternative models fail?

To understand why alternatives fail, it is necessary to keep in mind the central assumption in our favored model: one class inactivates completely while the other does not. This assumption, combined with the observed 4-to-1 ratio of inactivating to steady, leads to the 4- (or 5-) to-1 current ratio. Instead, any assumption of equal current will require partial inactivation of both classes of channels. Partial inactivation entails reversibility, reaching a steady distribution of closed, open, and inactivated at the end of a pulse. In this process, inactivation necessarily invades channels that did not open, which makes the alternatives incapable of matching the quantal aspects of inactivation embodied in the rule *suppression* = *decay*.

For a second level of explanation, we present programs that find the evolution of flux by deterministic methods, instead of averaging replicas of stochastic simulations. The temporal evolution of these models (state occupancies as a function of time) is determined by the transition rate constants and the initial conditions (Note that Szentesi et al., 2000, use deterministic with a different purpose—to qualify a feature of the observed inactivation, see main text). The system of differential equations is listed in text.

**Figure AC4.**
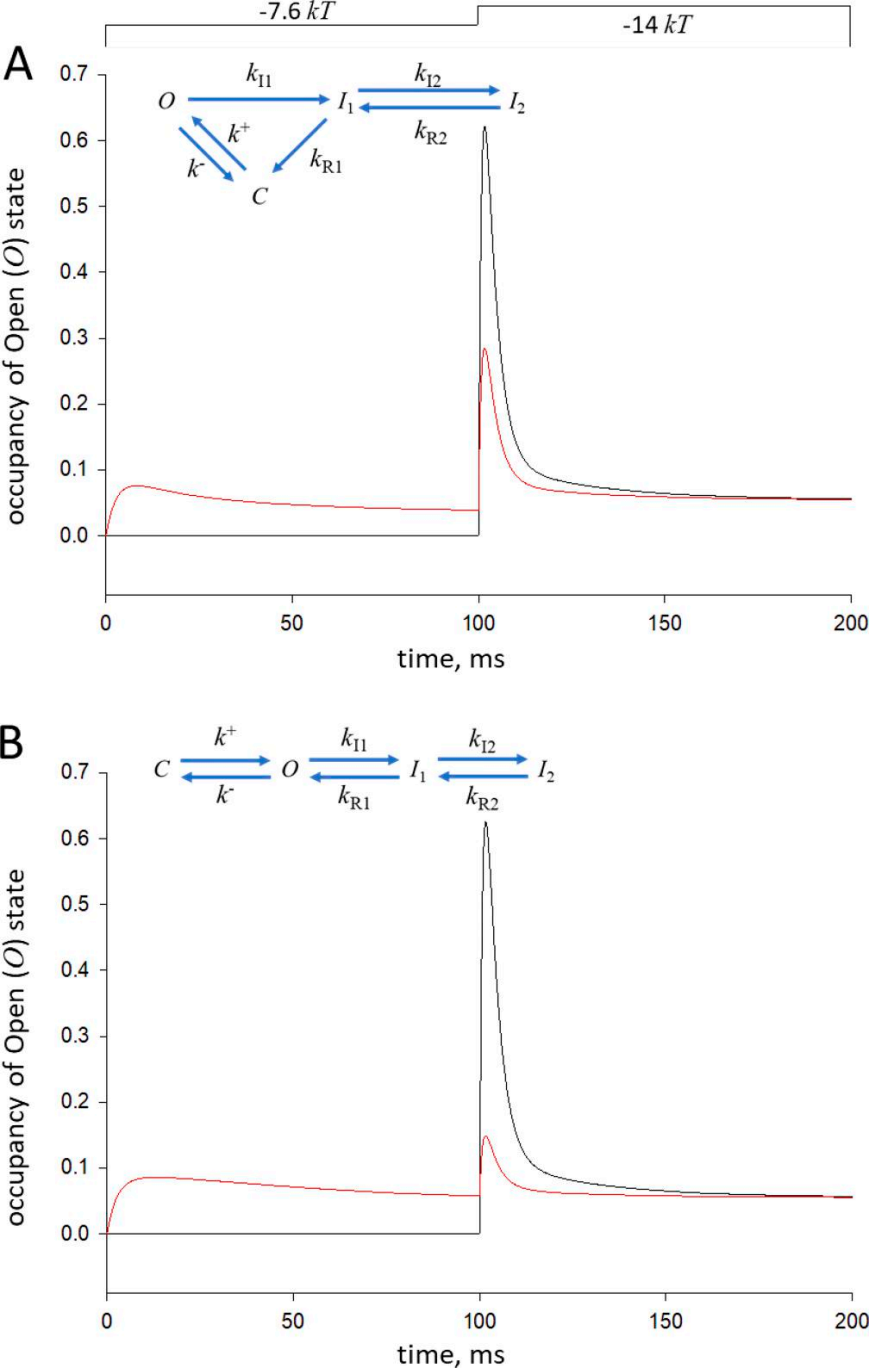
**Deterministic solutions comparable with the average flux in examples AC2 and AC3. (A)** The transients in A correspond to the model as represented in the state diagram in the same panel; they are solutions of the system of equations in Box AC1. **(B)** The transients in panel B are the corresponding solutions of the system of equations that describe the modified state model represented in the same panel. The simplified energy pulse pattern is represented at top. 7.6 kT is the electrical energy required for half activation of the C−O transition (*kT* = 1 in Eq. 13).

The graphs in Fig. AC4 illustrate the numerical solutions (Occupancy of state O as a function of time) of two simple 4-state models identical to those applied to V channels in the stochastic simulations of Figs. AC2 and AC3. At variance with those models, there are no C channels and no allosteric effects. The *C–O* rate constants vary with voltage identically as in the stochastic model of the main text. Namely the equilibrium constant for opening is an exponential function of the added electrical energy $\varepsilon_{\text{VC}}$

$$K_{V}=Ke^{-\varepsilon_{V}}$$

and the unidirectional transition rates are

$$k_{V}^{+}=k^{+}e^{-\nu_{V}\varepsilon_{V}}\;\text{and}\;k_{V}^{-}=k^{-}e^{-\left(1-\nu_{V}\right)\varepsilon_{V}}$$

which in this test are applied with v = 0.5.

The state diagrams are identical to the alternatives in the previous simulations (Figs. AC2 and AC3). Panels AC4, A and B, display the occupancies under a double-pulse protocol (with no interpulse interval, for simplicity of the calculations). The conditioning pulse is set at exactly the energy of half activation, calculated as the exponent in Eq. 13 that makes $K_{V}$ = 1. Because the “resting” value of K = 2,000^−1^ (Table 1), the half activation energy is 7.6 kT. The test pulse is at the saturation level, 14 kT. The graphs display both the double-pulse response and the test pulse response without conditioning.

The simulations show a conspicuous excess of suppression over decay and establish it to be a property of the voltage operated system, independent of the allosteric incorporation of other channels. Note that the model with *I*_*1*_ → *C* recovery works better than the one where inactivation recovers to O (although it does not come close to match the observed equality of decay and suppression). This is consistent with the interpretation that the equality requires “fatality” in the transition from open to inactivated (only and all open channels must inactivate). The first model keeps some of that property.

### AC.4: Models with multiple open states

**Figure AC5.**
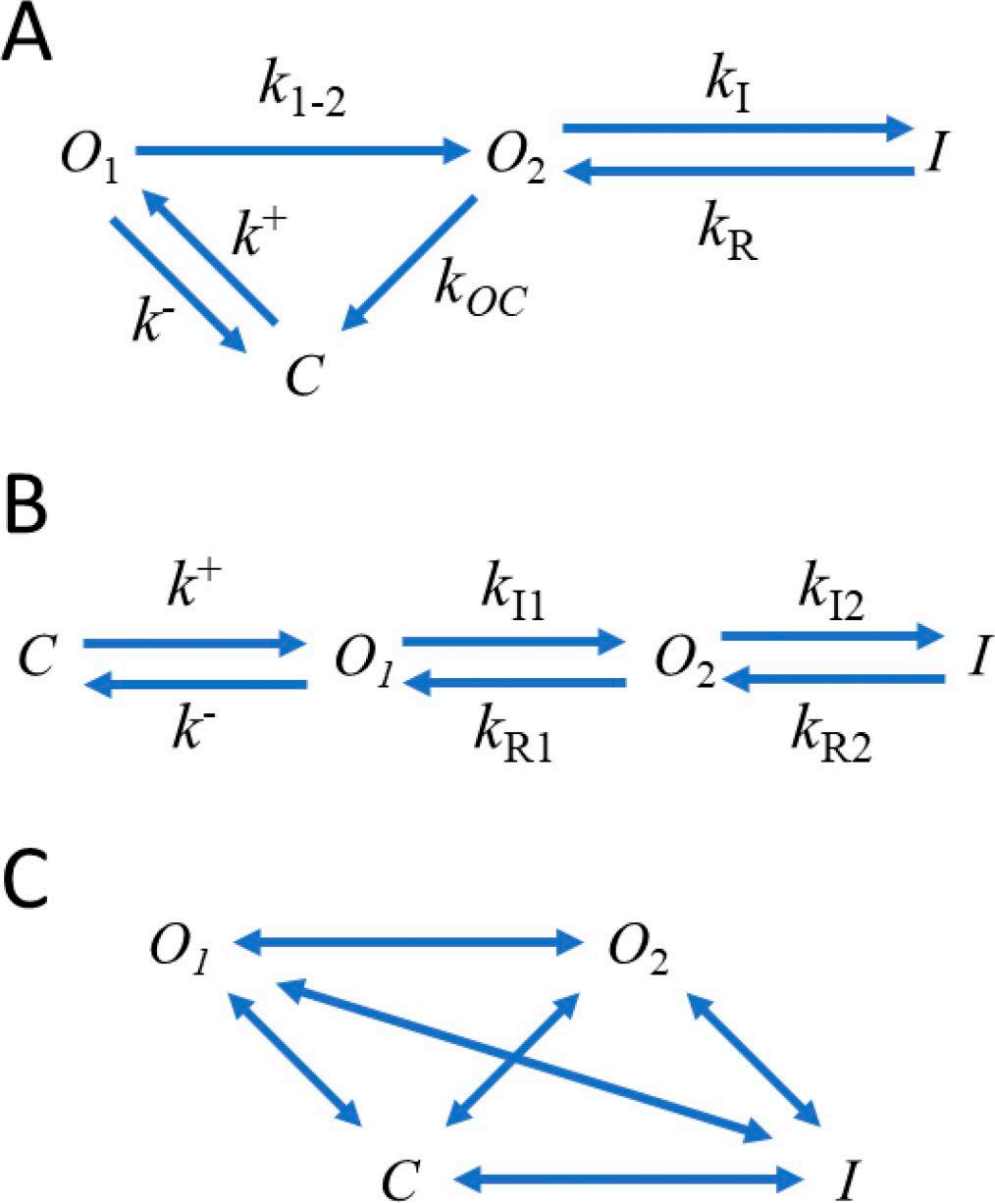
**Models with substates.** Channels V activate by voltage; otherwise, V and C have the same properties. **(A and B)** Similar to models in Figs. AC2 and AC3, with a sub-conductance open state $O_{2}$ substituted for $I_{1}$. **(C)** A generic four-state diagram to illustrate its 12 potential unidirectional transitions.

To complete the consideration of alternatives, we examine as possibilities the models with diagrams in Fig. AC5. These retain the functional identity of C and V channels, i.e., no difference is assumed between their open channel conductances. Instead, the conductances, for both channels, may be multiple. This expansion of possibilities allows, for example, to view the decay from peak to steady flux, in both C and V channels, as transitions from a fully open to a substate of smaller conductance, say 0.20 or 0.25 of the full value.

The stochastic simulations of schemes A and B failed similarly, and for the very reasons that those in Figs. AC2 and AC3 did, namely, inability to reproduce the quantal rule for inactivation (*suppression* = *decay*). Intuitively this should be easy to understand, as the inactivation to $I_{1}$ is replaced by a transition to a state of much lower conductance, resulting in little change from the examples AC2 and AC3. The rate constants in the models are constrained by the observations, so that the space of possible parameter values is limited and does not provide a way out of these failures.

The examinations above in no way exhaust the universe of possible schemes and assumptions. A next variation could test the schemes represented by diagram C in Fig. AC5, which keeps the assumption of four states but allows every possible transition. These generalized schemes have two problems. One is the addition of parameters—four-state schemes already require 12 transition rates, as well as assumptions for the states’ conductances, parameters for which there is no guidance. More damningly, the assumption of multiple open states of the same channel worsens the perplexing assumption of different physiological open currents in C and V. That the two or more conducting states be a property of the same channel is even less credible, as there have been no observations, in bilayers or *in situ*, of Ca^2+^-induced transitions to subconductances.

While far from proving anything, these exercises provide an additional appreciation of the unique merits of the simple model favored in the main article.
